# Genetic influences on suicide attempt in adolescence: Evaluating mediation by impulsivity and painful and provocative events

**DOI:** 10.1002/jcv2.70019

**Published:** 2025-06-05

**Authors:** Mallory Stephenson, Séverine Lannoy, Alexis C. Edwards

**Affiliations:** ^1^ Virginia Institute for Psychiatric and Behavioral Genetics Department of Psychiatry Virginia Commonwealth University Richmond Virginia USA

**Keywords:** adolescence, genetics, impulsivity, interpersonal‐psychological theory of suicide, suicide attempt

## Abstract

**Background:**

Genetic risk factors, impulsivity, and exposure to painful and provocative events (PPEs) have each been linked with risk for suicide attempt (SA). However, the degree to which genetic associations with SA are mediated by dimensions of impulsivity and PPEs remains unexplored, particularly in early adolescence.

**Methods:**

Participants were 6402 individuals (52.0% male, 48.0% female, 72.3% European ancestry, 27.7% African ancestry, mean age at baseline = 9.47 years, SD = 0.51 years) from the Adolescent Brain Cognitive Development (ABCD) Study. Genetic liability for SA was measured using polygenic scores and family history density scores. Multiple dimensions of impulsivity were assessed using self‐report measures and laboratory tasks, and potential PPEs included injuries, traumatic events, non‐suicidal self‐injury, and operations. A series of mediation models was specified to evaluate whether genetic associations with SA risk were mediated by impulsivity and PPE exposure. Separate models were tested in adolescents of European and African ancestry. Sex, age, socioeconomic factors, and depressive symptoms were included as covariates.

**Results:**

Genetic liability for SA was largely unrelated to impulsivity, PPE exposure, and SA risk (|β| = 0.00–0.34). In addition, there was little support for the hypothesis that more impulsive individuals are more likely to experience PPEs, with the exception that urgency and low conscientiousness were significantly related to non‐suicidal self‐injury (|β| = 0.09–0.19). Several dimensions of impulsivity and two PPEs (non‐suicidal self‐injury and traumatic events) were related to increased risk for SA (|β| = 0.32–0.76).

**Conclusions:**

Impulsivity and PPEs each contribute to risk for SA. However, there is little support for the hypothesis that genetic influences on SA are mediated by impulsivity and PPE exposure in early adolescence.

## Graphical abstract

Impulsivity and exposure to painful and provocative events each contribute to risk for suicide attempt. However, there is little support for the hypothesis that genetic influences on suicide attempt are mediated by impulsivity and painful and provocative event exposure in early adolescence.

**Key points**

- The hypothesis that genetic influences on suicide attempt (SA) may be mediated by impulsivity and exposure to painful and provocative events (PPEs) remains untested.
- Within a longitudinal, population‐based sample of adolescents, we found little evidence to suggest that genetic liability for SA is associated with impulsivity, PPEs, or risk for SA.
- Impulsivity and PPEs each contributed to risk for SA. However, the association between impulsivity and suicidal behavior was only observed in adolescents of European ancestry (EA).
- These findings highlight the need to develop more powerful genetic predictors of adolescent suicidal behavior, to consider multiple dimensions of impulsivity and PPE exposure, and to investigate genetic pathways to SA in individuals of different age groups and from underrepresented populations.

## INTRODUCTION

Adolescence is a critical period for the onset of suicidal thoughts and behaviors (Bolger et al., 1989; Nock et al., 2008, 2013). The lifetime prevalence of suicidal ideation (SI) and suicide attempt (SA) in youth has been estimated as 16% and 6%, respectively (Meter et al., 2023). Suicide rates in young people have also increased substantially over the past 15 years (Curtin & Garnett, 2023), underscoring the need to further investigate the development of suicidal behavior in this age group.

The Interpersonal‐Psychological Theory of Suicide (IPT) (Joiner, 2005; Van Orden et al., 2010) proposes that the development of suicidal thoughts and behaviors occurs via distinct but related processes. Within this theory, suicidal desire (or SI) is primarily shaped by thwarted belongingness and perceived burdensomeness. Thwarted belongingness occurs when someone's psychological need for social connection is unmet, while perceived burdensomeness is characterized by feelings of self‐hate or that one is a liability to others. The IPT proposes that an individual will experience SI if thwarted belongingness is accompanied by perceived burdensomeness, along with a feeling of hopelessness that the situation will improve. The transition from suicide ideation to attempt is then influenced by an individual's level of capability for suicide, which is acquired over time through exposure to physically painful and/or fear‐inducing experiences, labeled as PPEs. Through repeated exposure to PPEs, an individual may habituate to the painful and fearful aspects of self‐harm. Increased pain tolerance and lowered fear of death, in turn, increase the likelihood that an individual will act upon thoughts of suicide.

The IPT has been widely tested in adults (Chu et al., 2017), and a growing number of studies have applied the IPT to adolescent populations. One review and meta‐analysis of adolescent‐focused studies identified measures of acquired capability and PPE exposure as important predictors of SA in adolescence, despite mixed evidence for the roles of thwarted belongingness and perceived burdensomeness in the development of SI (Stewart et al., 2017). For example, a study conducted by King et al. (2024) demonstrated that greater exposure to PPEs is prospectively related to risk for SA in adolescence. A number of individual PPEs, such as non‐suicidal self‐injury (Barzilay et al., 2015; Klonsky et al., 2013), injection drug use (Liu et al., 2014), and health risk behaviors (Barzilay et al., 2015), have also been associated with adolescent SA.

Despite substantive empirical support for the IPT, one hypothesis forwarded by this theory remains untested: namely, that individuals who are genetically predisposed to impulsive behavior are more likely to experience PPEs, which in turn increases the likelihood that they will act upon suicidal thoughts (Van Orden et al., 2010). Investigating the mechanisms through which individuals are exposed to PPEs and develop the capability for suicidal behavior is important, both to evaluate the predictive utility of the IPT and to identify prevention targets. Further, studying this question in early adolescence offers the opportunity to elucidate the roles of genetic risk, impulsivity, and PPE exposure prior to the median age of onset of most psychiatric disorders (potentially relevant confounders) (Solmi et al., 2022).

In the present study, we used mediation models to test the hypothesis that genetic influences on SA are mediated by impulsivity and PPE exposure. Our analyses addressed several knowledge gaps. First, we examined the degree to which genetic associations with SA are mediated by impulsivity and exposure to PPEs in adolescence, as this developmental phase is characterized by particularly high levels of impulsivity (Casey et al., 2008) and is a key period for SA onset (Bolger et al., 1989; Nock et al., 2008, 2013). Yet, most studies on the IPT have been conducted in adults (Chu et al., 2017), and very few have included adolescent participants from racial and ethnic minority groups. Second, we leveraged the availability of summary statistics from genome‐wide association studies of suicidal behavior (Docherty et al., 2023) to construct indices of aggregate genetic liability for SA in adolescents of both European and African ancestry. Previous genetically‐informed studies of adolescent SA have been restricted to EA individuals (Lee et al., 2022; Loughnan et al., 2022), limiting our understanding of genetic pathways to SA in youth from other racial and ethnic backgrounds. Third, we considered several different impulsivity measures in our analyses, including a multi‐dimensional self‐report measure of impulsive personality traits and behavioral measures of response inhibition and delay discounting. Self‐report and task‐based measures of impulsivity are weakly correlated, can be linked with distinct neural mechanisms, and demonstrate unique associations with risk behavior (Cyders & Coskunpinar, 2011; Dalley & Robbins, 2017; Dang et al., 2020; Sharma et al., 2014). Therefore, triangulation across measures may offer insight into which dimensions of impulsivity are related to PPE exposure and SA risk. Fourth, because the IPT proposes that impulsivity and exposure to PPEs are primarily associated with the transition from suicidal thoughts to behavior, we also conducted sensitivity analyses limited to adolescents with a lifetime history of SI.

## METHODS

### Participants

Data were from the Adolescent Brain Cognitive Development (ABCD) Study, a longitudinal, multisite study of nearly 12,000 adolescents in the United States. Participants were invited to join the study at ages 9–10 years and are assessed approximately annually on a number of social, emotional, cognitive, and behavioral outcomes (Volkow et al., 2018). We used ABCD Release 5.0 (https://nda.nih.gov/study.html?id=2147), which includes data through the 3‐year follow‐up visit. The present analyses were limited to individuals who did not report a SA at baseline, had genetic data available, and were of empirically‐derived European or African ancestry. Individuals who reported a SA at baseline (*n* = 94) were excluded to ensure that the SA outcome did not precede the measurement of impulsivity and PPEs. If data were available on multiple individuals from the same family, one family member was randomly selected for inclusion in the analysis. The final analytic sample included 6402 individuals (48.0% female). Data were drawn from the baseline assessment (mean age = 9.47 years, SD = 0.51 years), 1‐year follow‐up assessment (mean age = 10.91 years, SD = 0.63 years), 2‐year follow‐up assessment (mean age = 12.02 years, SD = 0.66 years), and 3‐year follow‐up assessment (mean age = 12.91 years, SD = 0.64 years). Data collection procedures were approved by a central Institutional Review Board at the University of California, San Diego for most ABCD research sites, with a few sites obtaining local Institutional Review Board approval (Auchter et al., 2018). Parents or guardians provided written informed consent, and adolescents assented before participation in the study.

### Measures

#### Genetic liability for suicide attempt

In view of prior evidence that phenotype‐ and genotype‐based measures of genetic liability account for largely independent variance in psychiatric outcomes (Krebs et al., 2023), we included two genetic instruments in our analyses: one derived from molecular genetic data and one derived from information on family history of suicidal behavior.

**Genetic data and polygenic scores (PGS).** The biospecimen collection and genotyping protocols for ABCD have been described previously (Fan et al., 2023; Uban et al., 2018). After quality controls, genotypic data were available for 11,666 individuals at ∼500,000 variants.

We performed empirical ancestry assignment using the method described by Peterson et al. (2017). See the Supporting Information for details. In total, 2170 individuals were assigned to African ancestry (AFR), 2909 individuals were admixed from the Americas (AMR), 188 individuals were East Asian (EAS), 5815 individuals were European (EUR), and 372 individuals were South Asian (SAS) (Figure S1). The concordance rate between ancestry assignment and parental report of their child's race and ethnicity was greater than 91.0% (Table S1).

PGS for SA (PGS_SA_) were constructed using PRS‐CSx (Ruan et al., 2022). European ancestry (EA) and African ancestry (AA) summary statistics were drawn from the largest genome‐wide association study of SA to‐date (Docherty et al., 2023), which included 43,871 SA cases and 915,025 ancestry‐matched controls. Cases were defined by a lifetime history of SA, with approximately 13% of cases having died by suicide. Notably, both genetic epidemiology studies and molecular genetic studies have demonstrated that the genetic underpinnings of SA and psychiatric disorders are overlapping, but partially distinct (Brent & Melhem, 2008; Docherty et al., 2023; Mullins et al., 2022). In Docherty et al. (2023), for example, the genetic correlation between major depression and SA was estimated as 0.68. Therefore, PGS_SA_ encompass a genetic component that is shared between SA and other psychiatric and non‐psychiatric traits (e.g., substance use, risk tolerance), as well as a genetic component that is specific to SA.

Within each ancestral group, we regressed PGS_SA_ on the first 10 ancestral principal components (see the Supporting Information for details on the calculation of principal components) to correct for population stratification. The standardized residuals were carried forward for subsequent analyses.

**Family history density (FHD) scores.** Parents were asked to report whether any blood relative of their child had ever attempted or died by suicide. FHD scores were calculated as the weighted sum of the number of family members with a history of SA or death (with parents and full siblings weighted 0.50 and grandparents, aunts, uncles, and half‐siblings weighted 0.25), divided by the total family size. Outliers (>3 SD from the mean) were winsorized, and FHD scores were standardized prior to analysis.

#### Impulsivity

Measures of impulsivity were drawn from the baseline and one‐year follow‐up assessments. Abbreviated descriptions of each measure are provided below. See the Supporting Information for details (Supplemental Methods), including information regarding assessment timing (Figure S2).

**Response inhibition.** Participants completed the Stop‐Signal Task at baseline (Logan, 1994). Stop signal reaction time (RT) was calculated using the integration method (Eagle et al., 2008; Logan & Cowan, 1984). We created a standardized score prior to the analysis, with higher values reflecting lower response inhibition (i.e., greater impulsivity).

**Delay discounting.** Two tasks were used to assess delay discounting. The cash choice task (Wulfert et al., 2002) was completed during the baseline assessment. Participants were asked, “Would you rather have $75 in three days or $115 in three months?” The decision to receive $75 in three days was coded as 1, and the decision to receive $115 in three months was coded as 0. The adjusting delay discounting task (Koffarnus & Bickel, 2014) was completed during the one‐year follow‐up assessment. We calculated the area under the empirical discounting function, which could range from 0.0 (steepest possible discounting) to 1.0 (no delay discounting) (Myerson et al., 2001). We converted these values to z‐scores, then multiplied the scores by −1, such that higher scores reflect greater delay discounting.

**Impulsive personality traits.** Participants completed a 20‐item abbreviated youth version of the UPPS‐P Impulsive Behavior Scale (Watts et al., 2020), which includes five 4‐item subscales to evaluate the following dimensions of impulsivity: negative urgency (the tendency to act impulsively when experiencing negative affect), lack of premeditation (the tendency to act without careful thought or planning), lack of perseverance (the inclination to give up before completing a task), sensation‐seeking (the tendency of seek out excitement), and positive urgency (the tendency to act impulsively when experiencing positive affect). This measure was developed for the ABCD study and demonstrates excellent structural, convergent, and discriminant validity (Watts et al., 2020). Response options were on a four‐point scale, ranging from 1 = “agree strongly” to 4 = “disagree strongly.” A mean score was computed for each subscale.

#### Painful and provocative events

Painful and provocative events were selected to be consistent with the IPT (Joiner, 2005) and included a range of injuries and traumatic events, as well as a history of non‐suicidal self‐injury and operations. To ensure that PPEs preceded or were concurrent with the SA outcome, measures were drawn from the baseline and one‐year follow‐up assessments. A full list of PPEs is shown in Table 1, and details regarding the measurement of each variable are provided in the Supporting Information (Supplemental Methods, Table S2).

**TABLE 1:** Descriptive statistics for the primary study variables.

	EA (*N* = 4628)			AA (*N* = 1774)		
	*M* (SD)/*n* (%)	Range	Missing (*n*)	*M* (SD)/*n* (%)	Range	Missing (*n*)
Genetic liability for SA						
PGS	0.00 (1.00)	−3.90–3.76	0	0.00 (1.00)	−3.25–2.58	0
FHD scores	0.01 (0.01)	0.00–0.06	179	0.00 (0.01)	0.00–0.06	132
Impulsivity						
Stop‐signal RT	300.48 (105.41)	21.00–718.79	126	316.94 (136.52)	4.83–718.79	84
Cash choice task	1760 (38.0%)	0–1	80	664 (37.4%)	0–1	50
Delay discounting AUC	0.44 (0.28)	0.01–0.99	1023	0.38 (0.28)	0.01–0.99	442
Negative urgency	8.39 (2.55)	4–16	3	8.72 (2.85)	4–16	3
Lack of planning	7.90 (2.34)	4–16	3	7.46 (2.57)	4–16	3
Lack of perseverance	7.08 (2.23)	4–16	3	6.97 (2.36)	4–16	3
Sensation‐seeking	9.96 (2.62)	4–16	3	9.62 (2.82)	4–16	3
Positive urgency	7.66 (2.77)	4–16	3	8.68 (3.20)	4–16	3
PPEs						
Non‐suicidal self‐injury	577 (12.5%)	0–1	0	189 (10.7%)	0–1	0
Head injury	0.15 (0.41)	0–2	0	0.12 (0.39)	0–2	1
Knocked unconscious	72 (1.6%)	0–1	0	19 (1.1%)	0–1	1
Traumatic brain injury	1.07 (0.32)	1–3	130	1.04 (0.23)	1–3	183
Operation	1012 (21.9%)	0–1	0	201 (11.3%)	0–1	1
Broken bones	0.28 (0.62)	0–3	0	0.12 (0.38)	0–2	1
Sprains	0.23 (0.58)	0–3	0	0.17 (0.51)	0–3	1
Stitches	0.30 (0.61)	0–3	0	0.20 (0.55)	0–3	1
Other serious wounds	0.07 (0.29)	0–2	0	0.05 (0.27)	0–2	1
Falls	0.28 (0.70)	0–4	0	0.41 (1.19)	0–7	1
Burns	119 (2.6%)	0–1	0	60 (3.4%)	0–1	1
Bruises	0.23 (1.11)	0–7	0	0.12 (0.62)	0–5	1
Broken teeth	0.09 (0.33)	0–2	0	0.06 (0.30)	0–2	1
Animal bites	148 (3.2%)	0–1	0	40 (2.3%)	0–1	1
Threatened with death	40 (0.9%)	0–1	59	18 (1.0%)	0–1	29
Witnessed violence in the home	259 (5.6%)	0–1	59	226 (12.7%)	0–1	29
Experienced violence	31 (0.7%)	0–1	59	41 (2.3%)	0–1	29
Exposed to violence	20 (0.4%)	0–1	59	66 (3.7%)	0–1	29
Sexual assault	95 (2.1%)	0–1	59	39 (2.2%)	0–1	29
Car accident	132 (2.9%)	0–1	59	111 (6.3%)	0–1	29
Other accident	215 (4.7%)	0–1	59	62 (3.5%)	0–1	29
Fire	107 (2.3%)	0–1	59	52 (2.9%)	0–1	29
Natural disaster	120 (2.6%)	0–1	59	38 (2.1%)	0–1	29
Outcome						
SA	41 (0.9%)	0–1	88	26 (1.5%)	0–1	102
Covariates						
Female sex	2185 (47.2%)	0–1	0	885 (49.9%)	0–1	1
Age at baseline	9.47 (0.50)	8–11	4	9.47 (0.51)	8–11	2
Parental education			1			4
11^th^ grade or less	21 (0.5%)	‐‐‐	‐‐‐	103 (5.8%)	‐‐‐	‐‐‐
12^th^ grade	160 (3.5%)	‐‐‐	‐‐‐	449 (25.3%)	‐‐‐	‐‐‐
Some college	864 (18.7%)	‐‐‐	‐‐‐	727 (41.0%)	‐‐‐	‐‐‐
College graduate	3582 (77.4%)	‐‐‐	‐‐‐	491 (27.7%)	‐‐‐	‐‐‐
Household income			206			260
Low (<$50,000)	540 (11.7%)	‐‐‐	‐‐‐	995 (56.1%)	‐‐‐	‐‐‐
Middle ($50,000‐$99,999)	1369 (29.6%)	‐‐‐	‐‐‐	329 (18.5%)	‐‐‐	‐‐‐
High (>$100,000)	2513 (54.3%)	‐‐‐	‐‐‐	190 (10.7%)	‐‐‐	‐‐‐
Financial difficulties	0.26 (0.83)	0–7	0	1.03 (1.50)	0–7	9
Depressive symptoms	0.41 (1.30)	0–8	61	0.65 (1.64)	0–8	56

Abbreviations: AA, African ancestry; AUC, area under the curve; EA, European ancestry; FHD, family history density; SA, suicide attempt; PGS, polygenic scores; PPEs, painful and provocative events; RT, reaction time; .

#### Suicidal ideation and suicide attempt

Suicidal ideation and suicide attempt were assessed via self‐report using the Kiddie Schedule for Affective Disorders and Schizophrenia for the DSM‐5, Present and Lifetime Version (KSADS‐PL‐5) (Kaufman et al., 1997). The KSADS‐PL‐5 is a semi‐structured interview that has been shown to generate reliable and valid psychiatric diagnoses in children ages 7–17 (Kaufman et al., 1997). Suicidal ideation was assessed using one item: “Was there ever a time when you thought about wanting to kill yourself?” Participants who responded affirmatively at any assessment were considered to have a lifetime history of SI. To assess SA, participants were asked, “Was there ever a time when you did something to try to kill yourself and actually made a SA?” Participants who responded affirmatively at any follow‐up assessment were coded as 1, and all others were coded as 0.

#### Covariates

**Sex and age.** The child's sex (0 = male, 1 = female) and age at the baseline assessment were included as covariates.

**Parental education.** Parents reported on their highest level of education, which was coded as 1 = 11^th^ grade or less, 2 = 12^th^ grade, 3 = some college or Associate's degree, and 4 = college graduate (Jaeger, 1997). A dummy‐coded variable was then created, with 12^th^ grade as the reference category. However, few individuals had completed 11^th^ grade or less, leading to problems with model convergence. As a result, the categories for 11^th^ grade or less and 12^th^ grade were combined in the final analyses.

**Household income.** At the baseline assessment, parents reported on their total household income for the past 12 months. Household income was categorized as low (<$50,000), middle ($50,000‐$99,999), and high ($100,000 or more), as has been done previously (Giddens et al., 2022). A dummy‐coded variable was created, with middle income as the reference category.

**Financial difficulties.** Parents reported on financial difficulties using seven items, which asked whether they or their immediate family had experienced a series of financial problems in the past 12 months. The number of financial difficulties was represented by a sum score.

**Depressive symptoms.** During the baseline assessment, participants reported on their lifetime history of depressive symptoms using the KSADS‐PL‐5 (Kaufman et al., 1997). Number of depressive symptoms was represented as a sum score with a possible range of 0–8. The DSM‐5 criterion for recurrent thoughts of death, SI, or suicide attempts was excluded to avoid overlap with the SA outcome.

Figure S2 provides a visual representation of the assessment timing.

### Statistical analysis

#### Preliminary analyses

Because multiple measures of impulsivity and PPEs were available, a split‐half exploratory factor analysis (EFA) and confirmatory factor analysis (CFA) approach was used for item reduction. We randomly split the full ABCD dataset (*N* = 11,868) in two. Within the first split‐half sample, we used parallel analysis (Horn, 1965) to determine the number of retained factors. We used the psych R package (Revelle, 2017) to conduct EFA with promax rotation. We then used the lavaan R package (Rosseel, 2012) to perform CFA in the first split‐half sample, requiring simple structure. We retained items with factor loadings greater than 0.30 and performed CFA in the second split‐half sample. The Tucker‐Lewis index (TLI), comparative fit index (CFI), root mean square error of approximation (RMSEA), and standardized root mean square residual (SRMR) were used to assess model fit.

Because several latent factors were needed to represent the underlying structure of impulsivity and PPEs, including all factors in the same mediation model led to problems with model convergence. To address this, we conducted a final CFA in the full sample and used the lavPredict function from the lavaan R package to extract factor scores (Rosseel, 2012). These factor scores were carried forward for further analyses.

#### Mediation models

**FIGURE 1:**
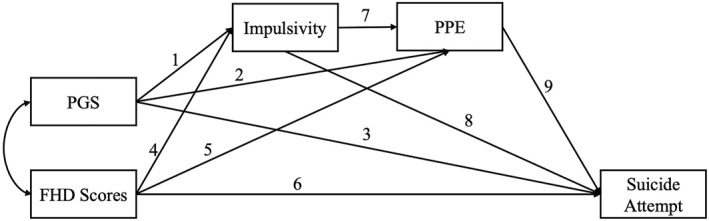
Path model used to investigate whether genetic influences on suicide attempt (SA) are mediated by impulsivity and exposure to painful and provocative events (PPEs). A separate model was specified for every possible combination of impulsivity and PPE measures. Sex, age, parental education, household income, financial difficulties, and depressive symptoms were included as covariates. Paths are numbered to correspond to their presentation in the Results. *Abbreviations.* PGS, polygenic scores; FHD, family history density; PPE, painful and provocative event.

Analyses were stratified by ancestry group. Within each subsample, we specified mediation models to evaluate whether the relationship between genetic liability for SA and risk for SA is mediated by impulsivity and PPEs (Figure 1). In light of the convergence issues described above, a separate model was tested for every possible combination of impulsivity and PPE factors. Sex, age, parental education, household income, financial difficulties, and depressive symptoms were included as covariates.

Analyses were conducted using the lavaan R package (Rosseel, 2012) with the weighted least square mean and variance adjusted estimator. Results are presented as standardized beta values with 95% confidence intervals. To correct for multiple testing, a false discovery rate (FDR)‐corrected *p*‐value less than 0.05 (Benjamini & Hochberg, 1995) was used as the threshold for statistical significance.

Missing data were handled using multiple imputation by chained equations, implemented in the mice R package (Buuren & Groothuis‐Oudshoorn, 2011). We imputed 25 datasets with 20 iterations for each imputation. We used the semTools R package (Jorgenson et al., 2022) to pool parameter estimates across datasets.

#### Sensitivity analyses

Because the IPT suggests that PPEs are primarily associated with the transition from SI to SA, we conducted a second set of mediation models within the subset of individuals who endorsed SI at any assessment (*N* = 639, 50.9% female, mean age at baseline = 9.47 years, SD = 0.50 years).

## RESULTS

### Preliminary factor analyses

#### Impulsivity

Correlations between measures of impulsivity are shown in Table S3. Parallel analysis indicated that three factors should be retained (Table S4). Lack of planning and lack of perseverance loaded most highly on Factor 1; stop‐signal RT, negative urgency, sensation‐seeking, and positive urgency loaded most highly on Factor 2; and performance on the cash choice and adjusted delay discounting tasks loaded most highly on Factor 3 (Table S5). When CFA was conducted in the first split‐half sample, stop‐signal RT and sensation‐seeking exhibited factor loadings below 0.30 (Table S6); these measures were examined separately in the primary analyses.

**FIGURE 2:**
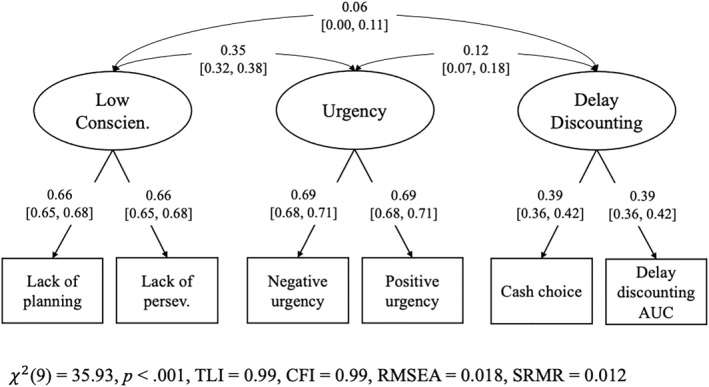
Confirmatory factor analysis (CFA) of impulsivity measures in the full sample. Stop‐signal reaction time (RT) and sensation‐seeking did not load highly on any latent factor and were examined separately in the primary analyses. Because each latent factor only had two indicators, factor loadings were constrained to be equal. Thresholds and residual variances are not shown. The variance of each latent factor was fixed to 1. AUC, area under the curve; Conscien., conscientiousness; CFI, comparative fit index; persev., perseverance; RMSEA, root mean square error of approximation; SRMR, standardized root mean square residual; TLI, Tucker‐Lewis index.

Confirmatory factor analysis in the second split‐half sample yielded adequate model fit, χ^2^(9) = 20.49, *p* = 0.015, TLI = 0.99, CFI = 0.99, RMSEA = 0.017, SRMR = 0.014. Therefore, we did not modify the model before conducting CFA in the full sample and calculating factor scores. Parameter estimates from CFA in the full sample are shown in Figure 2. We referred to Factor 1 as “low conscientiousness,” Factor 2 as “urgency,” and Factor 3 as “delay discounting.”

#### Painful and provocative events

Correlations among PPEs are shown in Table S7. Parallel analysis indicated that four factors should be retained (Table S8). Non‐suicidal self‐injury; being threatened with death; witnessing violence among grown‐ups in the home; experiencing violence; exposure to war, terrorism, and violence; sexual assault; car accidents; other significant accidents; fire; and natural disaster loaded most highly on Factor 1. Head injury, being knocked unconscious, and traumatic brain injury loaded most highly on Factor 2; operations, stitches, other serious wounds, broken teeth, and animal bites loaded most highly on Factor 3; and broken bones, sprains, falls, burns, and bruises loaded most highly on Factor 4 (Table S9). When CFA was conducted in the first split‐half sample, non‐suicidal self‐injury, operations, broken teeth, animal bites, and burns exhibited factor loadings less than 0.30 (Table S10). Non‐suicidal self‐injury and operations were examined separately in the primary analyses. Because broken teeth, animal bites, and burns were relatively rare, we did not consider these PPEs further.

**FIGURE 3:**
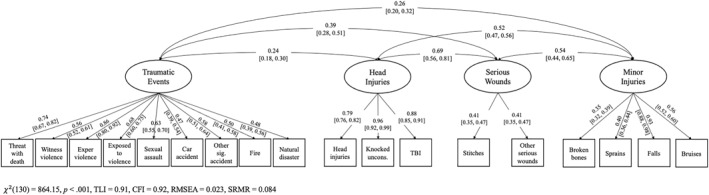
Confirmatory factor analysis (CFA) of painful and provocative events (PPEs) in the full sample. Non‐suicidal self‐injury and operations did not load highly on any latent factor and were examined separately in the primary analyses. Thresholds and residual variances are not shown. The variance of each latent factor was fixed to 1. Because Factor 3 (“serious wounds”) only had two indicators, the factor loadings were constrained to be equal. Threat, threatened; exper, experienced; sig., significant; uncons., unconscious; CFI, comparative fit index; RMSEA, root mean square error of approximation; SRMR, standardized root mean square residual; TBI, traumatic brain injury; TLI, Tucker‐Lewis index

Next, we performed CFA in the second split‐half sample, which yielded adequate model fit, *χ* ^2^(130) = 509.54, *p* < 0.001, TLI = 0.91, CFI = 0.93, RMSEA = 0.023, SRMR = 0.098. We did not modify the model before conducting CFA in the full sample and calculating factor scores. Parameter estimates from CFA in the full sample are shown in Figure 3. We referred to Factor 1 as “traumatic events,” Factor 2 as “head injuries,” Factor 3 as “serious wounds,” and Factor 4 as “minor injuries.”

### Descriptive statistics

Descriptive statistics for each of the study variables are shown in Table 1. Correlations between variables are shown in Tables S11 and S12 for EA and AA individuals, respectively.

### Mediation models

For impulsivity, three factor scores and two measured variables were included in the primary analyses: low conscientiousness, urgency, delay discounting, low response inhibition (i.e., stop‐signal reaction time), and sensation‐seeking. For PPEs, four factor scores and two measured variables were included in the primary analyses: traumatic events, head injuries, serious wounds, minor injuries, non‐suicidal self‐injury, and operations. We specified a separate model for every possible combination of impulsivity and PPE measures, which resulted in a total of 30 mediation models within each ancestral group. We used the Benjamini‐Hochberg procedure (Benjamini & Hochberg, 1995) to control the false discovery rate at 0.05.

#### Models in European ancestry individuals

Path coefficients from the mediation models in EA individuals are presented in Table 2. Below, we describe results based on the numbered paths depicted in Figure 1.

**TABLE 2:** Mediation model parameter estimates in individuals of European ancestry (EA).

	Traumatic events	Head injuries	Serious wounds	Minor injuries	NSSI	Operations
	β [95% CI]	β [95% CI]	β [95% CI]	β [95% CI]	β [95% CI]	β [95% CI]
Low conscientiousness						
Path 1: IMP ∼ PGS	0.01 [−0.16, 0.19]	0.02 [−0.16, 0.18]	0.01 [−0.09, 0.11]	0.01 [−0.09, 0.12]	0.01 [−0.09, 0.12]	0.01 [−0.09, 0.12]
Path 2: IMP ∼ FHD	0.05 [−0.12, 0.21]	0.05 [−0.12, 0.21]	0.05 [−0.05, 0.14]	0.05 [−0.05, 0.14]	0.05 [−0.06, 0.15]	0.05 [−0.06, 0.15]
Path 3: PPE ∼ PGS	0.07 [−0.25, 0.39]	−0.01 [−0.49, 0.46]	0.05 [−0.30, 0.40]	0.06 [−0.29, 0.41]	0.05 [−0.08, 0.19]	0.03 [−0.09, 0.15]
Path 4: PPE ∼ FHD	0.25 [−0.01, 0.51]	0.04 [−0.36, 0.45]	0.03 [−0.33, 0.39]	0.13 [−0.22, 0.48]	0.05 [−0.08, 0.17]	0.02 [−0.10, 0.14]
Path 5: PPE ∼ IMP	0.00 [−0.24, 0.23]	0.01 [−0.22, 0.24]	0.02 [−0.27, 0.31]	0.04 [−0.25, 0.32]	0.15 [0.04, 0.25]	0.00 [−0.10, 0.10]
Path 6: SA ∼ PGS	0.08 [−0.45, 0.62]	0.10 [−0.42, 0.63]	0.11 [−0.20, 0.43]	0.10 [−0.20, 0.41]	0.08 [−0.23, 0.39]	0.10 [−0.21, 0.42]
Path 7: SA ∼ FHD	0.06 [−0.44, 0.56]	0.14 [−0.36, 0.65]	0.15 [−0.15, 0.44]	0.14 [−0.15, 0.43]	0.12 [−0.17, 0.41]	0.14 [−0.15, 0.43]
Path 8: SA ∼ IMP	**0.58 [0.33, 0.82]**	**0.58 [0.35, 0.80]**	**0.58 [0.43, 0.73]**	**0.58 [0.44, 0.71]**	**0.52 [0.37, 0.66]**	**0.58 [0.44, 0.71]**
Path 9: SA ∼ PPE	**0.33 [0.23, 0.42]**	−0.09 [−3.91, 3.74]	−0.21 [−0.70, 0.29]	0.00 [−0.07, 0.08]	0.39 [0.03, 0.75]	0.08 [−0.33, 0.49]
Urgency						
Path 1: IMP ∼ PGS	0.02 [−0.14, 0.18]	0.02 [−0.14, 0.18]	0.02 [−0.07, 0.12]	0.02 [−0.07, 0.12]	0.02 [−0.08, 0.12]	0.02 [−0.07, 0.12]
Path 2: IMP ∼ FHD	0.04 [−0.12, 0.19]	0.04 [−0.12, 0.19]	0.04 [−0.05, 0.12]	0.04 [−0.05, 0.12]	0.04 [−0.06, 0.13]	0.04 [−0.05, 0.13]
Path 3: PPE ∼ PGS	0.07 [−0.24, 0.38]	−0.01 [−0.48, 0.45]	0.05 [−0.29, 0.38]	0.06 [−0.28, 0.39]	0.05 [−0.08, 0.18]	0.03 [−0.09, 0.15]
Path 4: PPE ∼ FHD	0.25 [−0.01, 0.51]	0.04 [−0.35, 0.44]	0.03 [−0.31, 0.37]	0.13 [−0.21, 0.47]	0.05 [−0.07, 0.17]	0.02 [−0.10, 0.13]
Path 5: PPE ∼ IMP	0.02 [−0.23, 0.28]	0.02 [−0.28, 0.32]	−0.01 [−0.31, 0.29]	0.03 [−0.26, 0.32]	**0.19 [0.08, 0.29]**	0.00 [−0.10, 0.10]
Path 6: SA ∼ PGS	0.07 [−0.45, 0.60]	0.09 [−0.42, 0.61]	0.10 [−0.20, 0.41]	0.10 [−0.20, 0.40]	0.08 [−0.22, 0.38]	0.09 [−0.21, 0.40]
Path 7: SA ∼ FHD	0.06 [−0.43, 0.54]	0.14 [−0.35, 0.64]	0.14 [−0.14, 0.42]	0.14 [−0.14, 0.41]	0.12 [−0.16, 0.40]	0.14 [−0.14, 0.42]
Path 8: SA ∼ IMP	**0.75 [0.53, 0.96]**	**0.76 [0.54, 0.97]**	**0.75 [0.62, 0.88]**	**0.75 [0.64, 0.86]**	**0.69 [0.56, 0.82]**	**0.75 [0.64, 0.87]**
Path 9: SA ∼ PPE	**0.32 [0.23, 0.42]**	−0.09 [−3.85, 3.67]	−0.21 [−0.68, 0.27]	0.00 [−0.07, 0.07]	0.34 [−0.01, 0.69]	0.08 [−0.32, 0.47]
Delay discounting						
Path 1: IMP ∼ PGS	−0.02 [−0.32, 0.27]	−0.02 [−0.31, 0.26]	−0.02 [−0.14, 0.09]	−0.02 [−0.13, 0.09]	−0.02 [−0.14, 0.09]	−0.02 [−0.14, 0.09]
Path 2: IMP ∼ FHD	0.01 [−0.28, 0.30]	0.01 [−0.28, 0.30]	0.01 [−0.10, 0.12]	0.01 [−0.10, 0.12]	0.01 [−0.10, 0.12]	0.01 [−0.10, 0.12]
Path 3: PPE ∼ PGS	0.07 [−0.21, 0.34]	−0.01 [−0.42, 0.40]	0.05 [−0.15, 0.24]	0.06 [−0.14, 0.25]	0.06 [−0.02, 0.13]	0.03 [−0.04, 0.10]
Path 4: PPE ∼ FHD	0.25 [0.03, 0.48]	0.05 [−0.30, 0.39]	0.03 [−0.17, 0.23]	0.13 [−0.07, 0.32]	0.05 [−0.01, 0.12]	0.02 [−0.05, 0.08]
Path 5: PPE ∼ IMP	0.02 [−0.09, 0.13]	0.01 [−0.10, 0.12]	0.03 [−0.05, 0.11]	0.02 [−0.06, 0.10]	0.01 [−0.02, 0.04]	0.00 [−0.03, 0.02]
Path 6: SA ∼ PGS	0.09 [−0.37, 0.55]	0.11 [−0.34, 0.56]	0.12 [−0.05, 0.30]	0.11 [−0.06, 0.28]	0.08 [−0.08, 0.25]	0.11 [−0.06, 0.28]
Path 7: SA ∼ FHD	0.08 [−0.34, 0.50]	0.17 [−0.26, 0.60]	0.17 [0.01, 0.33]	0.16 [0.01, 0.32]	0.14 [−0.02, 0.29]	0.16 [0.01, 0.32]
Path 8: SA ∼ IMP	0.05 [−0.12, 0.22]	0.06 [−0.11, 0.23]	0.06 [0.00, 0.13]	0.06 [−0.01, 0.12]	0.05 [−0.01, 0.12]	0.06 [−0.01, 0.12]
Path 9: SA ∼ PPE	**0.33 [0.26, 0.40]**	−0.09 [−3.40, 3.23]	−0.21 [−0.49, 0.07]	0.00 [−0.04, 0.04]	**0.51 [0.32, 0.69]**	0.08 [−0.14, 0.30]
Low response inhibition						
Path 1: IMP ∼ PGS	0.00 [−0.13, 0.13]	0.00 [−0.13, 0.13]	0.00 [−0.07, 0.07]	0.00 [−0.07, 0.07]	0.00 [−0.07, 0.07]	0.00 [−0.07, 0.07]
Path 2: IMP ∼ FHD	−0.01 [−0.14, 0.12]	−0.01 [−0.14, 0.12]	−0.01 [−0.08, 0.06]	−0.01 [−0.08, 0.06]	−0.01 [−0.08, 0.06]	−0.01 [−0.08, 0.06]
Path 3: PPE ∼ PGS	0.07 [−0.23, 0.36]	−0.01 [−0.46, 0.43]	0.05 [−0.25, 0.34]	0.06 [−0.24, 0.35]	0.06 [−0.06, 0.17]	0.03 [−0.07, 0.13]
Path 4: PPE ∼ FHD	0.25 [0.01, 0.49]	0.05 [−0.33, 0.43]	0.03 [−0.27, 0.33]	0.13 [−0.17, 0.42]	0.05 [−0.05, 0.16]	0.02 [−0.08, 0.12]
Path 5: PPE ∼ IMP	−0.03 [−0.31, 0.26]	−0.01 [−0.29, 0.27]	0.06 [−0.23, 0.36]	0.05 [−0.24, 0.34]	0.00 [−0.11, 0.11]	0.02 [−0.08, 0.11]
Path 6: SA ∼ PGS	0.09 [−0.41, 0.58]	0.11 [−0.39, 0.61]	0.12 [−0.14, 0.39]	0.11 [−0.15, 0.37]	0.08 [−0.18, 0.34]	0.11 [−0.16, 0.37]
Path 7: SA ∼ FHD	0.09 [−0.37, 0.54]	0.17 [−0.30, 0.65]	0.18 [−0.07, 0.42]	0.17 [−0.07, 0.41]	0.14 [−0.10, 0.38]	0.17 [−0.07, 0.41]
Path 8: SA ∼ IMP	0.52 [0.17, 0.88]	0.51 [0.16, 0.87]	**0.53 [0.33, 0.72]**	**0.51 [0.33, 0.70]**	**0.51 [0.32, 0.70]**	**0.51 [0.33, 0.70]**
Path 9: SA ∼ PPE	**0.33 [0.25, 0.42]**	−0.08 [−3.70, 3.53]	−0.21 [−0.63, 0.21]	0.00 [−0.06, 0.06]	**0.51 [0.23, 0.79]**	0.07 [−0.27, 0.41]
Sensation‐seeking						
Path 1: IMP ∼ PGS	0.00 [−0.12, 0.13]	0.00 [−0.11, 0.12]	0.00 [−0.03, 0.03]	0.00 [−0.03, 0.03]	0.00 [−0.03, 0.03]	0.00 [−0.03, 0.03]
Path 2: IMP ∼ FHD	0.01 [−0.11, 0.13]	0.01 [−0.11, 0.12]	0.01 [−0.02, 0.04]	0.01 [−0.02, 0.04]	0.01 [−0.02, 0.04]	0.01 [−0.02, 0.04]
Path 3: PPE ∼ PGS	0.07 [−0.20, 0.34]	−0.01 [−0.41, 0.38]	0.05 [−0.08, 0.17]	0.06 [−0.07, 0.18]	0.06 [0.01, 0.10]	0.03 [−0.01, 0.07]
Path 4: PPE ∼ FHD	0.25 [0.03, 0.48]	0.04 [−0.29, 0.38]	0.03 [−0.10, 0.16]	0.13 [0.00, 0.25]	0.05 [0.01, 0.10]	0.02 [−0.02, 0.06]
Path 5: PPE ∼ IMP	−0.01 [−0.27, 0.25]	0.13 [−0.12, 0.38]	0.05 [−0.07, 0.18]	0.13 [−0.01, 0.26]	0.00 [−0.05, 0.04]	−0.01 [−0.05, 0.03]
Path 6: SA ∼ PGS	0.09 [−0.37, 0.54]	0.11 [−0.32, 0.54]	0.12 [0.01, 0.23]	0.11 [0.00, 0.22]	0.08 [−0.02, 0.19]	0.11 [0.00, 0.22]
Path 7: SA ∼ FHD	0.08 [−0.33, 0.50]	0.17 [−0.24, 0.58]	**0.17 [0.07, 0.27]**	**0.17 [0.07, 0.26]**	**0.14 [0.04, 0.23]**	**0.16 [0.07, 0.26]**
Path 8: SA ∼ IMP	0.02 [−0.36, 0.40]	0.03 [−0.54, 0.59]	0.03 [−0.07, 0.12]	0.01 [−0.07, 0.10]	0.02 [−0.07, 0.10]	0.02 [−0.07, 0.11]
Path 9: SA ∼ PPE	**0.33 [0.26, 0.40]**	−0.09 [−3.28, 3.11]	−0.21 [−0.39, −0.03]	0.00 [−0.02, 0.03]	**0.51 [0.40, 0.62]**	0.08 [−0.06, 0.21]

*Note*: Results are presented as beta values with 95% confidence intervals. Statistically significant parameter estimates (FDR−corrected *p* < 0.05) are shown in bold font. Due to space limitations, only the path coefficients of central interest are shown. Paths are numbered to correspond to their presentation in the Results and Figure 1.

CI, confidence interval; FHD, family history density scores; IMP, impulsivity; PGS, polygenic scores; PPE, painful and provocative event; SA, suicide attempt.

**Originating from PGS**_**SA**_ **(Paths 1–3).** The associations between PGS_SA_ and dimensions of impulsivity (Path 1) were non‐significant, and effect sizes were small (|β| = 0.00–0.03). PGS_SA_ were not significantly associated with PPEs (Path 2, |β| = 0.01–0.07) or risk for SA (Path 3, |β| = 0.07–0.12).

**Originating from FHD (Paths 4–6).** Family history density scores were not significantly related to impulsivity (Path 4, |β| = 0.01–0.05) or PPEs (Path 5, |β| = 0.02–0.25). However, FHD scores were associated with elevated risk for SA (Path 6, |β| = 0.06–0.18).

**Originating from Impulsivity (Paths 7–8).** Higher levels of urgency were associated with increased likelihood of engaging in non‐suicidal self‐injury (β = 0.19). In all other models, the path from impulsivity to PPE exposure was not significant (|β| = 0.00–0.15). Lower conscientiousness, higher levels of urgency, and lower response inhibition were associated with higher risk for SA (|β| = 0.51–0.76). Delay discounting and sensation‐seeking were not significantly related to SA (|β| = 0.01–0.06).

**Originating from PPEs (Path 9).** Traumatic events and non‐suicidal self‐injury were associated with elevated SA risk (|β| = 0.32–0.51). Head injuries, serious wounds, minor injuries, and operations were not significantly associated with SA (|β| = 0.00–0.21).

#### Models in African ancestry individuals

Path coefficients from the mediation models in AA individuals are presented in Table 3.

**TABLE 3:** Mediation model parameter estimates in individuals of African ancestry.

	Traumatic events	Head injuries	Serious wounds	Minor injuries	NSSI	Operations
	β [95% CI]	β [95% CI]	β [95% CI]	β [95% CI]	β [95% CI]	β [95% CI]
Low conscientiousness						
Path 1: IMP ∼ PGS	−0.02 [−0.10, 0.06]	−0.02 [−0.35, 0.31]	−0.02 [−0.09, 0.05]	−0.02 [−0.09, 0.05]	−0.02 [−0.09, 0.05]	−0.02 [−0.09, 0.05]
Path 2: IMP ∼ FHD	0.03 [−0.05, 0.11]	0.03 [−0.29, 0.36]	0.03 [−0.04, 0.11]	0.03 [−0.04, 0.11]	0.03 [−0.04, 0.10]	0.03 [−0.04, 0.10]
Path 3: PPE ∼ PGS	−0.10 [−0.25, 0.05]	−0.01 [−1.12, 1.10]	0.04 [−0.15, 0.23]	0.08 [−0.14, 0.29]	−0.05 [−0.15, 0.04]	0.02 [−0.06, 0.11]
Path 4: PPE ∼ FHD	**0.34 [0.20, 0.48]**	0.31 [−0.27, 0.88]	0.04 [−0.15, 0.23]	0.07 [−0.15, 0.29]	0.06 [−0.02, 0.14]	0.09 [0.01, 0.16]
Path 5: PPE ∼ IMP	0.03 [−0.08, 0.14]	0.00 [−0.33, 0.32]	0.06 [−0.08, 0.20]	−0.05 [−0.21, 0.11]	**0.09 [0.03, 0.15]**	−0.01 [−0.07, 0.05]
Path 6: SA ∼ PGS	0.06 [−0.19, 0.30]	0.05 [−0.92, 1.03]	0.05 [−0.17, 0.27]	0.06 [−0.16, 0.28]	0.07 [−0.14, 0.28]	0.05 [−0.16, 0.26]
Path 7: SA ∼ FHD	0.08 [−0.09, 0.25]	0.09 [−0.60, 0.79]	0.09 [−0.06, 0.24]	0.09 [−0.06, 0.24]	0.06 [−0.08, 0.21]	0.07 [−0.07, 0.22]
Path 8: SA ∼ IMP	0.09 [−0.02, 0.20]	0.09 [−0.35, 0.53]	0.09 [−0.01, 0.18]	0.09 [−0.01, 0.19]	0.06 [−0.04, 0.15]	0.09 [0.00, 0.18]
Path 9: SA ∼ PPE	0.03 [−0.03, 0.09]	−0.02 [−0.53, 0.50]	0.03 [0.00, 0.06]	−0.05 [−0.09, −0.01]	**0.37 [0.20, 0.53]**	0.18 [−0.02, 0.37]
Urgency						
Path 1: IMP ∼ PGS	−0.05 [−0.12, 0.03]	−0.05 [−0.36, 0.27]	−0.05 [−0.12, 0.02]	−0.05 [−0.12, 0.02]	−0.05 [−0.11, 0.02]	−0.05 [−0.11, 0.02]
Path 2: IMP ∼ FHD	0.00 [−0.08, 0.07]	0.00 [−0.31, 0.31]	0.00 [−0.07, 0.07]	0.00 [−0.07, 0.07]	0.00 [−0.07, 0.06]	0.00 [−0.07, 0.06]
Path 3: PPE ∼ PGS	−0.09 [−0.24, 0.05]	−0.01 [−1.11, 1.10]	0.04 [−0.15, 0.23]	0.07 [−0.15, 0.29]	−0.05 [−0.14, 0.05]	0.02 [−0.06, 0.11]
Path 4: PPE ∼ FHD	**0.34 [0.20, 0.48]**	0.31 [−0.27, 0.88]	0.04 [−0.15, 0.23]	0.07 [−0.15, 0.29]	0.07 [−0.01, 0.15]	0.09 [0.01, 0.16]
Path 5: PPE ∼ IMP	0.10 [−0.01, 0.20]	0.01 [−0.38, 0.41]	−0.04 [−0.19, 0.11]	−0.09 [−0.26, 0.08]	**0.16 [0.10, 0.23]**	−0.02 [−0.08, 0.05]
Path 6: SA ∼ PGS	0.06 [−0.18, 0.30]	0.05 [−0.92, 1.03]	0.05 [−0.17, 0.27]	0.06 [−0.17, 0.28]	0.07 [−0.14, 0.28]	0.05 [−0.16, 0.26]
Path 7: SA ∼ FHD	0.08 [−0.09, 0.25]	0.10 [−0.60, 0.79]	0.09 [−0.06, 0.24]	0.09 [−0.06, 0.25]	0.07 [−0.08, 0.21]	0.08 [−0.07, 0.22]
Path 8: SA ∼ IMP	0.06 [−0.07, 0.20]	0.07 [−0.48, 0.61]	0.07 [−0.05, 0.19]	0.06 [−0.06, 0.19]	0.01 [−0.12, 0.13]	0.07 [−0.05, 0.19]
Path 9: SA ∼ PPE	0.03 [−0.03, 0.09]	−0.02 [−0.53, 0.50]	0.03 [0.00, 0.06]	−0.05 [−0.09, −0.01]	**0.38 [0.20, 0.55]**	0.18 [−0.02, 0.38]
Delay discounting						
Path 1: IMP ∼ PGS	0.03 [−0.10, 0.16]	0.03 [−0.49, 0.55]	0.03 [−0.09, 0.14]	0.03 [−0.09, 0.15]	0.03 [−0.08, 0.14]	0.03 [−0.08, 0.14]
Path 2: IMP ∼ FHD	0.00 [−0.13, 0.13]	0.00 [−0.53, 0.54]	0.00 [−0.11, 0.12]	0.00 [−0.12, 0.12]	0.00 [−0.11, 0.12]	0.00 [−0.11, 0.12]
Path 3: PPE ∼ PGS	−0.10 [−0.25, 0.05]	−0.01 [−1.11, 1.09]	0.04 [−0.14, 0.23]	0.08 [−0.14, 0.29]	−0.06 [−0.15, 0.04]	0.02 [−0.06, 0.11]
Path 4: PPE ∼ FHD	**0.34 [0.20, 0.48]**	0.31 [−0.26, 0.88]	0.04 [−0.15, 0.23]	0.07 [−0.15, 0.28]	0.07 [−0.01, 0.15]	0.09 [0.01, 0.16]
Path 5: PPE ∼ IMP	−0.01 [−0.07, 0.05]	−0.01 [−0.25, 0.24]	−0.06 [−0.14, 0.03]	−0.01 [−0.11, 0.08]	−0.04 [−0.07, 0.00]	0.00 [−0.04, 0.04]
Path 6: SA ∼ PGS	0.06 [−0.18, 0.29]	0.05 [−0.92, 1.02]	0.05 [−0.16, 0.27]	0.06 [−0.16, 0.27]	0.07 [−0.13, 0.28]	0.05 [−0.16, 0.25]
Path 7: SA ∼ FHD	0.08 [−0.08, 0.25]	0.10 [−0.59, 0.79]	0.09 [−0.06, 0.24]	0.09 [−0.06, 0.25]	0.07 [−0.07, 0.21]	0.08 [−0.07, 0.22]
Path 8: SA ∼ IMP	−0.07 [−0.15, 0.01]	−0.07 [−0.40, 0.26]	−0.07 [−0.14, 0.00]	−0.07 [−0.15, 0.00]	−0.06 [−0.13, 0.02]	−0.07 [−0.14, 0.00]
Path 9: SA ∼ PPE	0.03 [−0.03, 0.09]	−0.02 [−0.53, 0.50]	0.03 [0.00, 0.06]	−0.05 [−0.09, −0.01]	**0.37 [0.20, 0.53]**	0.18 [−0.02, 0.37]
Low response inhibition						
Path 1: IMP ∼ PGS	−0.01 [−0.06, 0.05]	−0.01 [−0.25, 0.23]	−0.01 [−0.06, 0.04]	−0.01 [−0.06, 0.04]	−0.01 [−0.06, 0.04]	−0.01 [−0.06, 0.04]
Path 2: IMP ∼ FHD	−0.01 [−0.06, 0.05]	−0.01 [−0.24, 0.23]	−0.01 [−0.06, 0.04]	−0.01 [−0.06, 0.04]	−0.01 [−0.06, 0.04]	−0.01 [−0.06, 0.04]
Path 3: PPE ∼ PGS	−0.10 [−0.24, 0.04]	−0.01 [−1.12, 1.10]	0.04 [−0.14, 0.22]	0.08 [−0.13, 0.29]	−0.06 [−0.15, 0.04]	0.02 [−0.06, 0.11]
Path 4: PPE ∼ FHD	**0.34 [0.20, 0.48]**	0.31 [−0.27, 0.88]	0.04 [−0.14, 0.22]	0.07 [−0.14, 0.28]	0.07 [−0.01, 0.14]	0.09 [0.01, 0.16]
Path 5: PPE ∼ IMP	0.01 [−0.14, 0.15]	0.04 [−0.41, 0.48]	0.02 [−0.17, 0.21]	0.04 [−0.17, 0.25]	0.06 [−0.02, 0.14]	−0.01 [−0.10, 0.07]
Path 6: SA ∼ PGS	0.05 [−0.18, 0.28]	0.05 [−0.93, 1.03]	0.05 [−0.16, 0.26]	0.06 [−0.16, 0.27]	0.07 [−0.13, 0.27]	0.05 [−0.15, 0.25]
Path 7: SA ∼ FHD	0.08 [−0.08, 0.24]	0.10 [−0.60, 0.79]	0.09 [−0.05, 0.24]	0.10 [−0.05, 0.24]	0.07 [−0.07, 0.20]	0.08 [−0.06, 0.22]
Path 8: SA ∼ IMP	0.12 [−0.02, 0.27]	0.13 [−0.50, 0.75]	0.12 [−0.01, 0.26]	0.13 [−0.01, 0.26]	0.10 [−0.03, 0.24]	0.13 [0.00, 0.26]
Path 9: SA ∼ PPE	0.03 [−0.02, 0.09]	−0.02 [−0.53, 0.50]	0.03 [0.00, 0.06]	−0.05 [−0.09, −0.01]	**0.37 [0.21, 0.53]**	0.18 [−0.01, 0.37]
Sensation−seeking						
Path 1: IMP ∼ PGS	0.01 [−0.04, 0.07]	0.01 [−0.23, 0.25]	0.01 [−0.04, 0.06]	0.01 [−0.04, 0.06]	0.01 [−0.04, 0.06]	0.01 [−0.04, 0.06]
Path 2: IMP ∼ FHD	0.01 [−0.05, 0.07]	0.01 [−0.24, 0.26]	0.01 [−0.04, 0.06]	0.01 [−0.04, 0.06]	0.01 [−0.04, 0.06]	0.01 [−0.04, 0.06]
Path 3: PPE ∼ PGS	−0.10 [−0.24, 0.04]	−0.01 [−1.12, 1.10]	0.04 [−0.14, 0.22]	0.07 [−0.13, 0.28]	−0.06 [−0.15, 0.03]	0.02 [−0.06, 0.11]
Path 4: PPE ∼ FHD	**0.34 [0.20, 0.48]**	0.31 [−0.27, 0.88]	0.04 [−0.14, 0.22]	0.07 [−0.14, 0.27]	0.07 [−0.01, 0.14]	0.09 [0.01, 0.16]
Path 5: PPE ∼ IMP	0.19 [0.05, 0.33]	−0.04 [−0.59, 0.52]	0.14 [−0.05, 0.33]	0.12 [−0.09, 0.34]	0.00 [−0.08, 0.08]	0.00 [−0.07, 0.08]
Path 6: SA ∼ PGS	0.05 [−0.18, 0.29]	0.05 [−0.93, 1.03]	0.05 [−0.16, 0.26]	0.05 [−0.16, 0.27]	0.07 [−0.12, 0.27]	0.05 [−0.15, 0.24]
Path 7: SA ∼ FHD	0.08 [−0.08, 0.24]	0.10 [−0.60, 0.79]	0.09 [−0.05, 0.23]	0.09 [−0.05, 0.24]	0.07 [−0.07, 0.20]	0.08 [−0.06, 0.21]
Path 8: SA ∼ IMP	−0.07 [−0.22, 0.08]	−0.07 [−0.68, 0.55]	−0.07 [−0.20, 0.06]	−0.06 [−0.19, 0.07]	−0.07 [−0.19, 0.06]	−0.07 [−0.19, 0.06]
Path 9: SA ∼ PPE	0.04 [−0.02, 0.10]	−0.02 [−0.53, 0.50]	0.03 [0.00, 0.06]	−0.05 [−0.09, −0.01]	**0.38 [0.22, 0.53]**	0.18 [−0.01, 0.36]

*Note*: Results are presented as beta values with 95% confidence intervals. Statistically significant parameter estimates (FDR‐corrected *p* < 0.05) are shown in bold font. Due to space limitations, only the path coefficients of central interest are shown. Paths are numbered to correspond to their presentation in the Results and Figure 1.

Abbreviations: CI, confidence interval; FHD, family history density scores; IMP, impulsivity; PGS, polygenic scores; PPE, painful and provocative event; SA, suicide attempt.

**Originating from PGS**_**SA**_ **(Paths 1–3).** PGS_SA_ were not related to impulsivity (Path 1, |β| = 0.01–0.05), PPE exposure (Path 2, |β| = 0.01–0.10), or SA risk (Path 3, |β| = 0.05–0.07).

**Originating from FHD (Paths 4–6).** FHD scores were not significantly associated with impulsivity (Path 4, |β| = 0.00–0.03). Higher FHD scores were associated with greater exposure to traumatic events (Path 5, β = 0.34), but associations with other PPEs were non‐significant (|β| = 0.04–0.31). FHD scores were not related to SA risk (Path 6, |β| = 0.06–0.10).

**Originating from Impulsivity (Paths 7–8).** Lower conscientiousness and greater urgency were associated with increased risk for non‐suicidal self‐injury (|β| = 0.09–0.16). All other associations between impulsivity and PPEs were non‐significant (|β| = 0.00–0.19). Impulsivity was not significantly associated with SA (Path 8, |β| = 0.01–0.13).

**Originating from PPEs (Path 9).** Non‐suicidal self‐injury was related to increased SA risk (|β| = 0.37–0.38). Exposure to other PPEs was not significantly associated with SA (|β| = 0.02–0.18).

### Sensitivity analyses

We conducted a second set of mediation models within the subsample of adolescents who endorsed suicidal thoughts (*N* = 639). Consistent with the primary analyses, separate models were tested in EA (*N* = 430) and AA (*N* = 209) adolescents.

#### Models in European ancestry individuals with suicidal ideation

Path coefficients from the mediation models in EA individuals with SI are presented in Table S13. The findings were similar to those observed in the primary analyses, with several exceptions. When the analyses were limited to individuals with SI, FHD scores were not significantly associated with SA risk (Path 6, |β| = 0.00–0.21), and urgency was no longer related to risk for non‐suicidal self‐injury (Path 7, β = 0.14). Lower response inhibition was still associated with SA risk (Path 8, |β| = 0.83–0.88), but low conscientiousness and urgency were not (|β| = 0.20–0.29). In addition, sensation‐seeking emerged as a significant predictor, such that individuals with SI who reported higher sensation‐seeking were more likely to attempt suicide (|β| = 0.53–0.58). Finally, Path 9, which represents the association between PPEs and SA, was statistically significant for traumatic events, serious wounds, and minor injuries (|β| = 0.19–0.24). The association between non‐suicidal self‐injury and SA was no longer significant when the analyses were limited to individuals with SI, but the effect sizes did not shift substantively (|β| = 0.31–0.42).

#### Models in African ancestry individuals with suicidal ideation

Path coefficients from the mediation models in AA individuals with SI are presented in Table S14. When the analyses were limited to those with SI, FHD scores for SA were related to lower response inhibition (|β| = 0.19–0.20). Family history density scores and low response inhibition were associated with elevated risk for head injuries (|β| = 0.58–0.75). Finally, minor injuries were related to risk for SA, in the negative direction (|β| = 0.09–0.10).

## DISCUSSION

Previous work has shown that suicidal behavior is moderately heritable (Edwards et al., 2021; Fu et al., 2002; Statham et al., 1998), related to multiple dimensions of impulsivity (Anestis et al., 2014; Liu et al., 2017; McHugh et al., 2019), and associated with a range of painful and fear‐inducing experiences (Khazem & Anestis, 2016; Mars et al., 2019; May & Klonsky, 2016; Vélez‐Grau et al., 2022; Walsh et al., 2021). Interpretations of the IPT suggest that each of these processes are inter‐related: Genetic factors may predispose an individual to be more impulsive, more impulsive individuals are more likely to be exposed to PPEs, and greater PPE exposure then increases one's capability for suicide (Van Orden et al., 2010). In the current study, we tested this hypothesis within a longitudinal, population‐based sample of adolescents. We focus on three key takeaways from the results of this study.

First, there was little evidence that PGS and FHD scores for SA were associated with impulsivity, PPEs, or SA. The only exceptions were positive associations between FHD scores and SA in EA adolescents, FHD scores and exposure to traumatic events in AA adolescents, and FHD scores and low response inhibition in AA adolescents with SI. All other associations with indices of genetic liability for SA were non‐significant. There are several potential explanations for this pattern of largely null results. One possibility is that our genetic instruments had insufficient predictive power. Because ABCD is a population‐based sample, the vast majority of adolescents (84%) had no family history of suicidal behavior, resulting in little variability in FHD scores. There is also evidence to suggest that measures of family history for SA have high sensitivity (97%) but relatively low specificity (44%) (Milne et al., 2009). Therefore, some suicide attempts in family members may have been missed. Further, PGS for SA account for ∼1% of the variance in suicidal behavior (Docherty et al., 2023; Mullins et al., 2022), though the estimated heritability of SA from twin studies ranges from 17% to 55% (Edwards et al., 2021; Fu et al., 2002; Statham et al., 1998). PGS_SA_ were also based on summary statistics from a genome‐wide association study conducted in mostly adult samples, and prior research indicates that there are qualitative differences between genetic factors influencing SA among young people versus adults (Edwards et al., 2021). The ABCD sample is quite young, and the current PGS_SA_ may become more informative in later waves. Another explanation is that perhaps a different set of genetic factors (i.e., not genetic liability for SA) contribute to the development of impulsivity and PPE exposure. Van Orden et al. (2010) highlight genetic predispositions to pain tolerance, fearlessness, and impulsivity (Arnatkeviciute et al., 2023; Sanchez‐Roige et al., 2019) as candidates for further research.

Second, within this sample of young adolescents, we found mixed support for the hypothesis that more impulsive individuals would experience more PPEs: Self‐report measures of impulsive personality traits were linked with non‐suicidal self‐injury, but not with injuries or operations. Behavioral measures of impulsivity were largely unrelated to PPE exposure, with the exception that low response inhibition was associated with head injuries in AA adolescents with SI. These findings underscore the importance of investigating impulsivity and PPEs as multidimensional constructs, suggesting that facets of impulsivity are not uniformly related to PPE exposure.

Third, impulsivity and PPE exposure both contributed to risk for SA in adolescence. However, different patterns of associations were observed depending on the specific PPE and dimension of impulsivity, genetic ancestry (which is highly correlated with race and ethnicity), and whether or not the analyses were limited to adolescents with a history of SI. For example, in EA adolescents, multiple dimensions of impulsivity were associated with elevated risk for SA, including low conscientiousness, urgency, and low response inhibition. In sensitivity analyses limited to EA adolescents with SI, low response inhibition, but not low conscientiousness or urgency, was statistically significantly related to SA, and sensation‐seeking also emerged as a significant predictor. On the other hand, there was no evidence to support a relationship between impulsivity and SA risk in AA adolescents, regardless of SI status. Of note, different patterns of statistical significance across groups does not necessarily mean that a particular risk factor (e.g., impulsivity) is more important in one group than another. Because PGS_SA_ were derived separately within each ancestral group, statistically comparing the magnitude of parameter estimates across ancestry is not feasible. Additional research is needed to empirically test for different pathways to SA across racial and ethnic groups and SI status.

When evaluating the relationship between PPEs and risk for SA, exposure to traumatic events (in EA adolescents only) and non‐suicidal self‐injury were associated with increased risk for SA, as has been observed previously (Grandclerc et al., 2016; Miché et al., 2020). Conversely, though the IPT suggests that physically painful experiences should predict increased SA risk, we observed several *negative* associations between PPEs and SA. For example, serious wounds were associated with lower SA risk in EA adolescents with SI, and minor injuries were associated with decreased risk for SA in AA adolescents with SI. These findings contrast with evidence for a positive effect of injuries on SA risk in adults (Sariaslan et al., 2023). Though speculative, one possibility is that, after an adolescent sustains an injury requiring medical attention, changes in parenting behaviors (e.g., increases in parental monitoring and warmth) or other features of the environment may protect against the transition to SA. However, these negative associations were inconsistently observed and warrant replication in other samples.

Lastly, it is notable that few statistically significant associations were observed between impulsivity, PPEs, and SA risk in AA adolescents. This may be partly driven by the smaller sample size (4628 EA adolescents vs. 1774 AA adolescents), which limited our statistical power to detect an effect in analyses of AA adolescents. However, an alternative explanation is that impulsivity and PPEs may be less relevant to the development of suicidal behavior in Black youth. One recent review found that only eight studies of the IPT included African American individuals, and none of those studies focused on adolescents (Robinson et al., 2022). This underscores the need for additional theoretically‐informed studies of suicidal behavior in Black adolescents, especially in view of the drastic increase in suicide rates in this group (Meza et al., 2022; Price & Khubchandani, 2019).

### Limitations

Our results should be interpreted in the context of several limitations. First, exposure to PPEs was assessed using lifetime measures, and it is possible that some events were experienced prior to the assessment of impulsivity. Second, we specified a series of mediation models with factor scores for impulsivity and PPEs, as opposed to including all latent factors in the same model. This approach was necessary to facilitate model convergence but does not account for measurement error in the factor scores, and standard errors may be underestimated. Third, though we included multiple self‐report and behavioral measures of impulsivity in our analyses, several dimensions of impulsivity remain unexamined, such as probabilistic discounting (Dalley & Robbins, 2017). Fourth, these analyses examine impulsivity and exposure to PPEs as distal risk factors for SA. This approach is consistent with the IPT, which suggests that individuals with elevated impulsivity may experience more PPEs over time, which in turn increases SA risk. However, other theoretical frameworks, such as the Integrated Motivational Volitional Model (O’Connor & Kirtley, 2018), propose that impulsivity plays a more proximal role in suicidal behavior, and there is empirical evidence to suggest that impulsivity and access to means are immediate precursors to self‐harm among adolescents (Townsend et al., 2016). Further, though facets of trait impulsivity (e.g., impulsive personality traits) are relatively stable, behavioral measures of impulsivity (e.g., response inhibition) may vary considerably over time. The present study cannot provide insight into the proximal processes through which state and trait impulsivity contribute to SA risk; this is an important area for future research.

## CONCLUSIONS

We investigated whether genetic influences on adolescent SA are mediated by impulsivity and exposure to PPEs. There was limited evidence to suggest that genetic risk for SA, dimensions of impulsivity, and exposure to PPEs are related to one another. Impulsivity and PPEs each contributed to SA risk, offering some preliminary evidence that adolescents with more impulsive personality traits, lower response inhibition, and greater PPE exposure (particularly engagement in non‐suicidal self‐injury) are at elevated risk for suicidal behavior and may benefit from targeted prevention efforts. Additional work is needed to improve polygenic prediction of suicidal behavior and to further investigate genetic pathways to SA in adolescence, particularly in underrepresented groups.

## AUTHOR CONTRIBUTIONS

**Mallory Stephenson**: Conceptualization; formal analysis; methodology; visualization; writing‐original draft. **Séverine Lannoy**: Conceptualization; writing‐review and editing. **Alexis C Edwards**: Conceptualization;supervision; writing‐review and editing.

## CONFLICT OF INTEREST STATEMENT

The authors declare no conflicts of interest.

## ETHICAL CONSIDERATIONS

Data collection procedures were approved by a central Institutional Review Board at the University of California, San Diego for most ABCD research sites, with a few sites obtaining local Institutional Review Board approval (Auchter et al., 2018). Parents or guardians provided written informed consent, and adolescents assented before participation in the study.

## Supporting information

Supplementary Material

## Data Availability

The data that support the findings from this study are available from the NDA (https://nda.nih.gov/). Restrictions apply to the availability of these data, which were used under license for this study.
