# Supplementary material for: Genetic influences on suicide attempt in adolescence: Evaluating mediation by impulsivity and painful and provocative events
Source: JCPP Adv. 2025 Jun 5;6(1):e70019. doi: 10.1002/jcv2.70019 (PMC12973133; doi:10.1002/jcv2.70019)
Supplement: Supplementary file 1 — Supplementary Material [file JCV2-6-e70019-s001.docx]

**Supporting Information:** Genetic influences on suicide attempt in adolescence: Evaluating mediation by impulsivity and painful and provocative events

**Table of Contents**

[**Supplemental Methods** 2](#_Toc184117129)

[**Supplemental References** 7](#_Toc184117130)

[Table S1. Concordance between parental report of their child’s race and ethnicity and genetic ancestry assignment by Mahalanobis distance. 11](#_Toc184117131)

[Table S2. Items used to evaluate exposure to traumatic events in the Adolescent Brain Cognitive Development Study. 12](#_Toc184117132)

[Table S3. Correlations among measures of impulsivity 13](#_Toc184117133)

[Table S4. Results from parallel analysis of measures of impulsivity 14](#_Toc184117134)

[Table S5. Factor loadings for exploratory factor analysis of impulsivity in the first split-half sample 15](#_Toc184117135)

[Table S6. Confirmatory factor analysis of impulsivity in the first split-half sample 16](#_Toc184117136)

[Table S7. Correlations between painful and provocative events 17](#_Toc184117137)

[Table S8. Results from parallel analysis of painful and provocative events 18](#_Toc184117138)

[Table S9. Factor loadings for exploratory factor analysis of painful and provocative events in the first split-half sample 19](#_Toc184117139)

[Table S10. Confirmatory factor analysis of painful and provocative events in the first split-half sample 22](#_Toc184117140)

[Table S11. Correlations between the primary study variables in European ancestry individuals 23](#_Toc184117141)

[Table S12. Correlations between the primary study variables in African ancestry individuals 24](#_Toc184117142)

[Table S13. Mediation model parameter estimates in European ancestry individuals with suicidal ideation. 25](#_Toc184117143)

[Table S14. Mediation model parameter estimates in African ancestry individuals with suicidal ideation. 27](#_Toc184117144)

[Figure S1. Assignment to super-populations in the Adolescent Brain Cognitive Development Study (ABCD). 29](#_Toc184117145)

[Figure S2. Diagrammatic representation of assessment timing. 30](#_Toc184117146)

# **Supplemental Methods**

**Ancestry Assignment**

We performed empirical ancestry assignment using the method described by Peterson et al. (2017). Briefly, variants from the 1000 Genomes Project (1KGP) phase 3 reference panel (2504 samples, 26 populations, 5 super-populations) were merged with the ABCD genotypes. Regions with high linkage disequilibrium were excluded (Price et al., 2006, 2008), and the common set of variants was pruned (r^2^ < 0.1) using PLINK 1.9 (Chang et al., 2015; Purcell et al., 2007), yielding 97,821 semi-independent variants. EIGENSOFT and SmartPCA (Patterson et al., 2006; Price et al., 2006) were used to perform principal component analysis using only the 1KGP reference panel. The solution was then projected onto the ABCD data to generate 10 principal components (PCs).

Using all 10 ancestry PCs, we calculated the median and variance for each of the 26 1KGP populations. Each ABCD sample was assigned to the 1KGP population with the minimum Mahalanobis distance, which was then used to determine their assignment to one of the five 1KGP super-populations: African (AFR), admixed from the Americas (AMR), East Asian (EAS), European (EUR), and South Asian (SAS). Multivariate outliers (> 3 SD from the super-population median, *n* = 212) were removed, leaving 2,170 individuals assigned to AFR, 2,909 assigned to AMR, 188 assigned to EAS, 5,815 assigned to EUR, and 372 assigned to SAS (Figure S1).

**Ancestral Principal Components**

Within each ancestral group, we calculated genetic PCs using PC-AiR, which allows for population structure inference in samples of related individuals (Conomos et al., 2015). We employed the same methods described by Fan et al. (2023) for trans-ancestry principal component analysis in ABCD.

**Imputation and Quality Control**

Samples were imputed to TOPMED (Taliun et al., 2021), as described previously (Fan et al., 2023). Within each ancestral group, we excluded variants with a minor allele frequency less than 0.1%, as well as variants that did not pass Hardy-Weinberg equilibrium (*p* < 1 x 10^-10^). In total, 13,549,177 variants and 26,853,183 variants passed these quality control thresholds and were available for analysis in the EUR and AFR sub-groups, respectively.

**Measurement of Impulsivity**

***Response Inhibition***

Participants completed the Stop-Signal Task during an fMRI scan at baseline (Logan, 1994). They were presented with a left- or right-pointing arrow and asked to press a button that corresponded to the direction of the arrow as quickly and accurately as possible. On stop signal trials, the participant was shown an arrow pointing up (stop signal) after the arrow pointing left or right (go signal) appeared. They were told to inhibit their response to the go signal when it was followed by a stop signal. This task was administered in 2 runs of 180 trials each (150 go and 30 stop trials). Go trials consisted of the presentation of a left- or right-pointing arrow for 1 second or until a response, whichever came first. Stop trials consisted of the presentation of a left- or right-pointing arrow for a Stop Signal Delay (SSD), followed by the presentation of a stop signal for 300 milliseconds. The SSD was varied to maintain ~50% success in stop trials (Casey et al., 2018). Stop signal reaction time was calculated using the integration method (Eagle et al., 2008; Logan & Cowan, 1984). We created a standardized score prior to the analysis, with higher values reflecting lower response inhibition (i.e., greater impulsivity).

***Delay Discounting***

The adjusting delay discounting task (Koffarnus & Bickel, 2014) was completed during the one-year follow-up assessment. Participants were given 42 choices between a small reward given immediately versus a larger $100 reward given at various points in the future. Choices were presented in seven randomly ordered blocks, which were defined by the time when the $100 reward would be given: 6 hours, 1 day, 1 week, 1 month, 3 months, 1 year, or 5 years. Within each block, the amount of the smaller immediate reward was adjusted based on the participant’s previous choice. This titration converged on an “indifference point,” where the smaller intermediate reward is considered equal in value to the delayed but larger reward. For each participant, we calculated the area under the empirical discounting function (Myerson et al., 2001). The area under the curve could range from 0.0 (steepest possible discounting) to 1.0 (no delay discounting). We converted these values to z-scores, then multiplied the scores by -1, such that higher scores reflect greater delay discounting.

**Measurement of Painful and Provocative Events (PPEs)**

***Non-Suicidal Self-Injury***

Non-suicidal self-injury was assessed using the Kiddie Schedule for Affective Disorders and Schizophrenia for the DSM-5, Present and Lifetime Version (KSADS-PL-5) (Kaufman et al., 1997), which was completed by both parents and adolescents. If either the parent or adolescent indicated that the adolescent had ever purposely hurt themselves, this variable was coded as 1.

***Injuries Requiring Medical Attention***

Parents were asked to report how many times their child had been to a doctor, a nurse, a nurse practitioner, the emergency room, or a clinic for the following reasons: a head injury, being knocked unconscious, a broken bone, a sprain, stitches, other serious wounds, a fall, a burn, a bruise, broken teeth, an animal bite, a gunshot wound, and a knife or other weapon wound. At the baseline assessment, questions focused on the child’s lifetime. At the one-year follow-up assessment, questions referred to the period since the last study visit. For each injury, we computed a prorated sum score across assessments.

Because very few adolescents had experienced a gunshot, knife, or other weapon wound in their lifetime (*n*s < 50 in the full ABCD sample), these variables were not considered in subsequent analyses. In addition, the remaining injury variables were highly skewed and kurtotic. To address this, we winsorized outliers (> 3 SD from the mean). Because this greatly restricted the range of each variable, we then treated each injury variable as ordinal in the analyses. For example, for head injuries, the possible range after winsorization was 0 to 2. Therefore, in subsequent analyses, we handled this as an ordinal variable, with possible values of 0 = no head injuries, 1 = one head injury in the child’s lifetime, and 2 = two or more head injuries in the child’s lifetime.

***Traumatic Brain Injury (TBI)***

Parents were interviewed using The Ohio State University TBI Identification Method (Bogner et al., 2017; Corrigan & Bogner, 2007), which was used to classify the child’s worst head injury as 1 = improbable TBI (no TBI or a TBI without any loss of consciousness or memory loss), 2 = possible mild TBI (a TBI with memory loss but no loss of consciousness), 3 = mild TBI (TBI with loss of consciousness for less than 30 minutes), 4 = moderate TBI (TBI with loss of consciousness for 30 minutes to 24 hours), or 5 = severe TBI (TBI with loss of consciousness for more than 24 hours). We took the maximum value across assessments. Because TBIs with loss of consciousness were rarely endorsed, we combined categories 3 through 5, such that 1 = improbable TBI, 2 = possible mild TBI, and 3 = TBI.

***Operations***

Parents reported on whether or not their child had ever been to a doctor for an operation, either in the child’s lifetime (baseline assessment) or since the last study visit (one-year follow-up assessment) (Todd et al., 2003). If parents responded affirmatively at either assessment, adolescents were coded as 1. All others were coded as 0.

***Traumatic Events***

Parents reported on their child’s lifetime exposure to traumatic events using the KSADS-PL-5 (Kaufman et al., 1997). For each event, if the parent responded affirmatively to any relevant item at either assessment, the adolescent was coded as 1. All others were coded as 0. In some cases, several items from the KSADS-PL-5 were grouped thematically to ensure an adequate sample size. The KSADS-PL-5 items used to evaluate exposure to each PPE are shown in Table S2.

# **Supplemental References**

Bogner, J. A., Whiteneck, G. G., MacDonald, J., Juengst, S. B., Brown, A. W., Philippus, A. M., Marwitz, J. H., Lengenfelder, J., Mellick, D., Arenth, P., & Corrigan, J. D. (2017). Test-retest reliability of traumatic brain injury outcome measures: A traumatic brain injury model systems study. *The Journal of Head Trauma Rehabilitation*, *32*(5), E1–E16. https://doi.org/10.1097/HTR.0000000000000291

Casey, B. J., Cannonier, T., Conley, M. I., Cohen, A. O., Barch, D. M., Heitzeg, M. M., Soules, M. E., Teslovich, T., Dellarco, D. V., Garavan, H., Orr, C. A., Wager, T. D., Banich, M. T., Speer, N. K., Sutherland, M. T., Riedel, M. C., Dick, A. S., Bjork, J. M., Thomas, K. M., … Dale, A. M. (2018). The Adolescent Brain Cognitive Development (ABCD) study: Imaging acquisition across 21 sites. *Developmental Cognitive Neuroscience*, *32*, 43–54. https://doi.org/10.1016/j.dcn.2018.03.001

Chang, C. C., Chow, C. C., Tellier, L. C., Vattikuti, S., Purcell, S. M., & Lee, J. J. (2015). Second-generation PLINK: Rising to the challenge of larger and richer datasets. *GigaScience*, *4*, 7. https://doi.org/10.1186/s13742-015-0047-8

Conomos, M. P., Miller, M., & Thornton, T. (2015). Robust inference of population structure for ancestry prediction and correction of stratification in the presence of relatedness. *Genetic Epidemiology*, *39*(4), 276–293. https://doi.org/10.1002/gepi.21896

Corrigan, J. D., & Bogner, J. (2007). Initial reliability and validity of the Ohio State University TBI Identification Method. *The Journal of Head Trauma Rehabilitation*, *22*(6), 318. https://doi.org/10.1097/01.HTR.0000300227.67748.77

Eagle, D. M., Baunez, C., Hutcheson, D. M., Lehmann, O., Shah, A. P., & Robbins, T. W. (2008). Stop-signal reaction-time task performance: Role of prefrontal cortex and subthalamic nucleus. *Cerebral Cortex (New York, N.Y.: 1991)*, *18*(1), 178–188. https://doi.org/10.1093/cercor/bhm044

Fan, C. C., Loughnan, R., Wilson, S., & Hewitt, J. K. (2023). Genotype data and derived genetic instruments of Adolescent Brain Cognitive Development Study for better understanding of human brain development. *Behavior Genetics*, *53*(3), 159–168. https://doi.org/10.1007/s10519-023-10143-0

Kaufman, J., Birmaher, B., Brent, D., Rao, U., Flynn, C., Moreci, P., Williamson, D., & Ryan, N. (1997). Schedule for affective disorders and schizophrenia for school-age children-present and lifetime version (K-SADS-PL): Initial reliability and validity data. *Journal of the American Academy of Child and Adolescent Psychiatry*, *36*(7), 980–988. https://doi.org/10.1097/00004583-199707000-00021

Koffarnus, M. N., & Bickel, W. K. (2014). A 5-trial adjusting delay discounting task: Accurate discount rates in less than 60 seconds. *Experimental and Clinical Psychopharmacology*, *22*(3), 222–228. https://doi.org/10.1037/a0035973

Logan, G. D. (1994). On the ability to inhibit thought and action: A users’ guide to the stop signal paradigm. In *Inhibitory processes in attention, memory, and language* (pp. 189–239). Academic Press.

Logan, G. D., & Cowan, W. B. (1984). On the ability to inhibit thought and action: A theory of an act of control. *Psychological Review*, *91*(3), 295–327. https://doi.org/10.1037/0033-295X.91.3.295

Patterson, N., Price, A. L., & Reich, D. (2006). Population structure and eigenanalysis. *PLoS Genetics*, *2*(12), e190. https://doi.org/10.1371/journal.pgen.0020190

Peterson, R. E., Edwards, A. C., Bacanu, S.-A., Dick, D. M., Kendler, K. S., & Webb, B. T. (2017). The utility of empirically assigning ancestry groups in cross-population genetic studies of addiction. *The American Journal on Addictions*, *26*(5), 494–501. https://doi.org/10.1111/ajad.12586

Price, A. L., Patterson, N. J., Plenge, R. M., Weinblatt, M. E., Shadick, N. A., & Reich, D. (2006). Principal components analysis corrects for stratification in genome-wide association studies. *Nature Genetics*, *38*(8), 904–909. https://doi.org/10.1038/ng1847

Price, A. L., Weale, M. E., Patterson, N., Myers, S. R., Need, A. C., Shianna, K. V., Ge, D., Rotter, J. I., Torres, E., Taylor, K. D., Goldstein, D. B., & Reich, D. (2008). Long-range LD can confound genome scans in admixed populations. *American Journal of Human Genetics*, *83*(1), 132–135. https://doi.org/10.1016/j.ajhg.2008.06.005

Purcell, S., Neale, B., Todd-Brown, K., Thomas, L., Ferreira, M. A. R., Bender, D., Maller, J., Sklar, P., de Bakker, P. I. W., Daly, M. J., & Sham, P. C. (2007). PLINK: A tool set for whole-genome association and population-based linkage analyses. *American Journal of Human Genetics*, *81*(3), 559–575. https://doi.org/10.1086/519795

Taliun, D., Harris, D. N., Kessler, M. D., Carlson, J., Szpiech, Z. A., Torres, R., Taliun, S. A. G., Corvelo, A., Gogarten, S. M., Kang, H. M., Pitsillides, A. N., LeFaive, J., Lee, S., Tian, X., Browning, B. L., Das, S., Emde, A.-K., Clarke, W. E., Loesch, D. P., … Abecasis, G. R. (2021). Sequencing of 53,831 diverse genomes from the NHLBI TOPMed Program. *Nature*, *590*(7845), Article 7845. https://doi.org/10.1038/s41586-021-03205-y

Todd, R. D., Joyner, C. A., Heath, A. C., Neuman, R. J., & Reich, W. (2003). Reliability and stability of a semistructured DSM-IV interview designed for family studies. *Journal of the American Academy of Child and Adolescent Psychiatry*, *42*(12), 1460–1468. https://doi.org/10.1097/00004583-200312000-00013

# Table S1. Concordance between parental report of their child’s race and ethnicity and genetic ancestry assignment by Mahalanobis distance.

| Race and Ethnicity | *n* | EUR | AMR | AFR | SAS | EAS | Concordance |
| --- | --- | --- | --- | --- | --- | --- | --- |
| White, non-Hispanic | 6208 | 5649 | 535 | 6 | 18 | 0 | 0.910 |
| Black | 1806 | 2 | 76 | 1728 | 0 | 0 | 0.956 |
| White, Hispanic | 1118 | 26 | 1091 | 1 | 0 | 0 | 0.976 |
| More than one race | 1362 | 105 | 570 | 384 | 273 | 30 |  |
| Other | 516 | 8 | 467 | 28 | 8 | 5 |  |
| Asian | 228 | 0 | 9 | 0 | 69 | 150 | 0.961 |
| Missing | 159 | 10 | 125 | 18 | 3 | 3 |  |
| Native American | 57 | 15 | 36 | 5 | 1 | 0 |  |
| *Total* | 11454 | 5815 | 2909 | 2170 | 372 | 188 |  |

*Abbreviations.* EUR = European; AMR = admixed from the Americas; AFR = African; SAS = South Asian; EAS = East Asian.

# Table S2. Items used to evaluate exposure to traumatic events in the Adolescent Brain Cognitive Development Study.

| **PPE** | **KSADS-PL-5 Item(s)** |
| --- | --- |
| Threatened with death | A family member threatened to kill your child |
|  | A non-family member threatened to kill your child |
| Witnessed violence among grown-ups in the home | Witnessed the grown-ups in the home push, shove, or hit one another |
| Experienced violence | Beaten to the point of having bruises by a grown-up in the home |
|  | Shot, stabbed, or brutally beaten by a grown-up in the home |
|  | Shot, stabbed, or brutally beaten by a non-family member |
| Exposure to war, terrorism, and violence | Witnessed or present during an act of terrorism |
|  | Witnessed death or mass destruction in a war zone |
|  | Witnessed someone shot or stabbed in the community |
| Sexual assault | A grown-up in the home touched your child in their privates, had your child touch their privates, or did other sexual things to your child |
|  | An adult outside your family touched your child in their privates, had your child touch their privates, or did other sexual things to your child |
|  | A peer forced your child to do something sexually |
| Car accident | A car accident in which your child or another person in the car was hurt bad enough to require medical attention |
| Other significant accident | Another significant accident for which your child needed specialized and intensive medical treatment |
| Fire | Witnessed or caught in a fire that caused significant property damage or personal injury |
| Natural disaster | Witnessed or caught in a natural disaster that caused significant property damage or personal injury |

*Abbreviations.* PPE = painful and provocative event; KSADS-PL-5 = Kiddie Schedule for Affective Disorders and Schizophrenia for the DSM-5, Present and Lifetime Version.

Table S3. Correlations among measures of impulsivity.

|  | **1** | **2** | **3** | **4** | **5** | **6** | **7** |
| --- | --- | --- | --- | --- | --- | --- | --- |
| 1. Stop-signal reaction time | 1 |  |  |  |  |  |  |
| 2. Cash choice task | -.04 | 1 |  |  |  |  |  |
| 3. Delay discounting AUC | -.01 | .12 | 1 |  |  |  |  |
| 4. Negative urgency | .05 | .01 | .03 | 1 |  |  |  |
| 5. Lack of planning | .03 | .01 | .01 | .16 | 1 |  |  |
| 6. Lack of perseverance | .06 | .00 | .02 | .13 | .45 | 1 |  |
| 7. Sensation-seeking | .01 | -.01 | -.02 | .14 | .06 | -.10 | 1 |
| 8. Positive urgency | .07 | .05 | .05 | .49 | .21 | .17 | .19 |

*Abbreviations.* AUC = area under the curve.

Table S4. Results from parallel analysis of measures of impulsivity.

| Rank | Eigenvalue | Minimum significant eigenvalue |
| --- | --- | --- |
| **1** | **1.859** | **1.061** |
| **2** | **1.247** | **1.038** |
| **3** | **1.153** | **1.021** |
| 4 | 0.994 | 1.007 |
| 5 | 0.879 | 0.993 |
| 6 | 0.853 | 0.978 |
| 7 | 0.522 | 0.961 |
| 8 | 0.494 | 0.940 |

*Note.* Retained factors are shown in bold font.

Table S5. Factor loadings for exploratory factor analysis of impulsivity in the first split-half sample.

|  |  | Factor Loading | 95% CI |
| --- | --- | --- | --- |
| Factor 1 | |  |  |
|  | Stop-signal reaction time | 0.05 | [0.02, 0.08] |
|  | Cash choice task | 0.02 | [-0.01, 0.04] |
|  | Delay discounting AUC | 0.01 | [-0.02, 0.03] |
|  | Negative urgency | 0.08 | [0.06, 0.11] |
|  | **Lack of planning** | **0.45** | **[0.42, 0.48]** |
|  | **Lack of perseverance** | **1.06** | **[1.03, 1.10]** |
|  | Sensation seeking | -0.12 | [-0.15, -0.09] |
|  | Positive urgency | 0.07 | [0.06, 0.09] |
| Factor 2 | |  |  |
|  | **Stop-signal reaction time** | **0.07** | **[0.03, 0.11]** |
|  | Cash choice task | 0.01 | [-0.02, 0.05] |
|  | Delay discounting AUC | 0.03 | [0.00, 0.07] |
|  | **Negative urgency** | **0.54** | **[0.49, 0.59]** |
|  | Lack of planning | 0.09 | [0.06, 0.12] |
|  | Lack of perseverance | -0.26 | [-0.28, -0.24] |
|  | **Sensation seeking** | **0.29** | **[0.25, 0.33]** |
|  | **Positive urgency** | **0.83** | **[0.76, 0.92]** |
| Factor 3 | |  |  |
|  | Stop-signal reaction time | -0.04 | [-0.10, 0.03] |
|  | **Cash choice task** | **0.46** | **[0.06, 0.99]** |
|  | **Delay discounting AUC** | **0.32** | **[0.03, 0.66]** |
|  | Negative urgency | 0.09 | [0.03, 0.20] |
|  | Lack of planning | 0.04 | [-0.02, 0.11] |
|  | Lack of perseverance | 0.02 | [0.00, 0.04] |
|  | Sensation seeking | -0.03 | [-0.10, 0.08] |
|  | Positive urgency | 0.15 | [0.08, 0.31] |

*Note.* Indicators that were carried forward for confirmatory factor analysis are shown in bold font. *Abbreviations.* AUC = area under the curve; CI = confidence interval.

Table S6. Confirmatory factor analysis of impulsivity in the first split-half sample.

|  |  | Factor Loading | 95% CI |
| --- | --- | --- | --- |
| Factor 1 | |  |  |
|  | Lack of planning | 0.65 | [0.63, 0.68] |
|  | Lack of perseverance | 0.65 | [0.63, 0.68] |
| Factor 2 | |  |  |
|  | *Stop-signal reaction time* | *0.11* | *[0.07, 0.14]* |
|  | Negative urgency | 0.57 | [0.52, 0.61] |
|  | *Sensation seeking* | *0.17* | *[0.14, 0.20]* |
|  | Positive urgency | 0.85 | [0.79, 0.92] |
| Factor 3 | |  |  |
|  | Cash choice task | 0.38 | [0.33, 0.43] |
|  | Delay discounting AUC | 0.38 | [0.33, 0.43] |

*Note.* Factor loadings were equated for factors with only two indicators. Indicators with factor loadings less than 0.30 are shown in italics. *Abbreviations.* AUC = area under the curve; CI = confidence interval.

Table S7. Correlations between painful and provocative events.

|  | **1** | **2** | **3** | **4** | **5** | **6** | **7** | **8** | **9** | **10** | **11** | **12** | **13** | **14** | **15** | **16** | **17** | **18** | **19** | **20** | **21** | **22** |
| --- | --- | --- | --- | --- | --- | --- | --- | --- | --- | --- | --- | --- | --- | --- | --- | --- | --- | --- | --- | --- | --- | --- |
| 1. NSSI | 1 |  |  |  |  |  |  |  |  |  |  |  |  |  |  |  |  |  |  |  |  |  |
| 2. Head injury | .06 | 1 |  |  |  |  |  |  |  |  |  |  |  |  |  |  |  |  |  |  |  |  |
| 3. Unconscious | -.01 | .56 | 1 |  |  |  |  |  |  |  |  |  |  |  |  |  |  |  |  |  |  |  |
| 4. TBI | .06 | .67 | .88 | 1 |  |  |  |  |  |  |  |  |  |  |  |  |  |  |  |  |  |  |
| 5. Operation | .09 | .07 | .02 | .03 | 1 |  |  |  |  |  |  |  |  |  |  |  |  |  |  |  |  |  |
| 6. Broken bones | .00 | .12 | .16 | .16 | .15 | 1 |  |  |  |  |  |  |  |  |  |  |  |  |  |  |  |  |
| 7. Sprain | .03 | .19 | .23 | .16 | .10 | .25 | 1 |  |  |  |  |  |  |  |  |  |  |  |  |  |  |  |
| 8. Stitches | .06 | .32 | .13 | .16 | .12 | .11 | .03 | 1 |  |  |  |  |  |  |  |  |  |  |  |  |  |  |
| 9. Other wounds | .03 | .32 | .15 | .14 | .11 | .07 | .11 | .17 | 1 |  |  |  |  |  |  |  |  |  |  |  |  |  |
| 10. Falls | .01 | .47 | .34 | .34 | .07 | .31 | .33 | .23 | .17 | 1 |  |  |  |  |  |  |  |  |  |  |  |  |
| 11. Burns | .05 | .07 | .06 | .08 | .04 | .03 | .10 | .09 | .38 | .16 | 1 |  |  |  |  |  |  |  |  |  |  |  |
| 12. Bruises | .01 | .22 | .15 | .14 | .04 | .08 | .23 | .11 | .19 | .55 | .25 | 1 |  |  |  |  |  |  |  |  |  |  |
| 13. Broken teeth | .02 | .07 | .04 | -.02 | .06 | .07 | .08 | .09 | .16 | .19 | .03 | .16 | 1 |  |  |  |  |  |  |  |  |  |
| 14. Animal bites | .03 | .08 | .11 | .04 | .09 | -.02 | .11 | .21 | .30 | .14 | .21 | .19 | .06 | 1 |  |  |  |  |  |  |  |  |
| 15. Threat with death | .15 | .17 | .16 | .03 | .12 | .07 | .12 | .04 | .06 | .08 | .03 | .04 | .02 | .07 | 1 |  |  |  |  |  |  |  |
| 16. Witnessed viol | .14 | .10 | .11 | .04 | .02 | -.02 | .07 | .08 | .10 | .13 | .08 | .17 | .00 | .03 | .43 | 1 |  |  |  |  |  |  |
| 17. Experienced viol | .15 | .04 | -.05 | -.03 | -.08 | .00 | .00 | -.02 | .04 | .16 | .00 | .14 | .02 | -.16 | .64 | .64 | 1 |  |  |  |  |  |
| 18. Exposed to viol | .11 | .09 | .01 | .01 | -.05 | -.03 | .01 | .03 | -.03 | .09 | .04 | .13 | .07 | .12 | .52 | .39 | .66 | 1 |  |  |  |  |
| 19. Sexual assault | .18 | .13 | .21 | .11 | .06 | .03 | .13 | .09 | .02 | .07 | -.03 | .07 | .04 | .04 | .57 | .43 | .47 | .39 | 1 |  |  |  |
| 20. Car accident | .09 | .16 | .01 | .15 | .02 | .09 | .06 | .05 | .07 | .13 | .17 | .10 | .06 | .01 | .30 | .27 | .30 | .30 | .21 | 1 |  |  |
| 21. Other accident | .05 | .26 | .28 | .28 | .15 | .38 | .10 | .26 | .35 | .22 | .26 | .10 | .10 | .17 | .33 | .15 | .30 | .28 | .26 | .33 | 1 |  |
| 22. Fire | .03 | .11 | .21 | .06 | .09 | .00 | .11 | .07 | .14 | .09 | .09 | -.05 | .06 | .12 | .39 | .19 | .32 | .36 | .31 | .25 | .24 | 1 |
| 23. Natural disaster | .04 | .09 | .05 | -.01 | .05 | .04 | .03 | .04 | .13 | .11 | -.12 | .07 | .01 | -.01 | .28 | .10 | .38 | .40 | .26 | .29 | .27 | .42 |

*Note.* Different correlation coefficients were estimated based on the variable type. *Abbreviations.* NSSI = non-suicidal self-injury; TBI = traumatic brain injury; threat = threatened with death; viol = violence.

Table S8. Results from parallel analysis of painful and provocative events.

| Rank | Eigenvalue | Minimum significant eigenvalue |
| --- | --- | --- |
| **1** | **4.215** | **2.549** |
| **2** | **3.230** | **2.092** |
| **3** | **1.954** | **1.721** |
| **4** | **1.504** | **1.454** |
| 5 | 1.268 | 1.319 |
| 6 | 1.210 | 1.238 |
| 7 | 1.183 | 1.180 |
| 8 | 1.120 | 1.134 |
| 9 | 0.995 | 1.094 |
| 10 | 0.957 | 1.058 |
| 11 | 0.898 | 1.025 |
| 12 | 0.778 | 0.993 |
| 13 | 0.743 | 0.959 |
| 14 | 0.670 | 0.924 |
| 15 | 0.542 | 0.888 |
| 16 | 0.517 | 0.845 |
| 17 | 0.459 | 0.797 |
| 18 | 0.374 | 0.733 |
| 19 | 0.320 | 0.646 |
| 20 | 0.249 | 0.478 |
| 21 | 0.177 | 0.191 |
| 22 | -0.089 | -0.040 |
| 23 | -0.274 | -0.277 |

*Note.* Retained factors are shown in bold font.

Table S9. Factor loadings for exploratory factor analysis of painful and provocative events in the first split-half sample.

|  |  | Factor Loading | 95% CI |
| --- | --- | --- | --- |
| Factor 1 | |  |  |
|  | **Non-suicidal self-injury** | **0.18** | **[-9.69, 10.87]** |
|  | Head injury | 0.10 | [-4.65, 5.42] |
|  | Knocked unconscious | -0.21 | [-17.22, 15.17] |
|  | Traumatic brain injury | 0.10 | [-3.22, 3.29] |
|  | Operation | 0.04 | [-3.25, 3.60] |
|  | Broken bones | 0.02 | [-4.10, 4.62] |
|  | Sprains | 0.00 | [-3.76, 4.24] |
|  | Stitches | 0.09 | [-6.35, 7.06] |
|  | Other serious wounds | 0.12 | [-5.00, 5.75] |
|  | Falls | -0.05 | [-5.51, 6.24] |
|  | Burns | -0.05 | [-15.46, 14.18] |
|  | Bruises | -0.18 | [-3.55, 3.78] |
|  | Broken teeth | -0.01 | [-4.89, 4.55] |
|  | Animal bites | -0.20 | [-10.88, 9.28] |
|  | **Threatened with death** | **0.79** | **[-38.20, 43.69]** |
|  | **Witnessed violence** | **0.51** | **[-22.54, 25.89]** |
|  | **Experienced violence** | **0.85** | **[-37.02, 42.71]** |
|  | **Exposure to violence** | **0.68** | **[-31.27, 35.81]** |
|  | **Sexual assault** | **0.63** | **[-27.26, 31.09]** |
|  | **Car accident** | **0.41** | **[-18.95, 21.71]** |
|  | **Other significant accident** | **0.42** | **[-18.22, 20.94]** |
|  | **Fire** | **0.54** | **[-18.87, 21.79]** |
|  | **Natural disaster** | **0.50** | **[-22.65, 25.88]** |
| Factor 2 | |  |  |
|  | Non-suicidal self-injury | 0.00 | [-3.89, 3.68] |
|  | **Head injury** | **0.51** | **[-25.65, 29.31]** |
|  | **Knocked unconscious** | **1.16** | **[-53.39, 61.00]** |
|  | **Traumatic brain injury** | **0.84** | **[-37.29, 42.96]** |
|  | Operation | -0.04 | [-2.20, 2.01] |
|  | Broken bones | 0.10 | [-7.92, 8.95] |
|  | Sprains | 0.08 | [-8.26, 9.34] |
|  | Stitches | 0.07 | [-6.53, 7.22] |
|  | Other serious wounds | 0.03 | [-6.97, 6.90] |
|  | Falls | 0.14 | [-17.74, 19.90] |
|  | Burns | -0.03 | [-13.98, 13.33] |
|  | Bruises | -0.13 | [-9.38, 10.24] |
|  | Broken teeth | -0.03 | [-5.51, 4.99] |
|  | Animal bites | -0.11 | [-4.85, 4.66] |
|  |  | Factor Loading | 95% CI |
|  | Threatened with death | -0.47 | [-25.67, 22.51] |
|  | Witnessed violence | -0.05 | [-4.37, 4.14] |
|  | Experienced violence | -0.03 | [-3.72, 3.33] |
|  | Exposure to violence | -0.42 | [-14.45, 12.08] |
|  | Sexual assault | 0.13 | [-1.41, 1.83] |
|  | Car accident | 0.05 | [-7.07, 6.80] |
|  | Other significant accident | 0.18 | [-4.24, 5.12] |
|  | Fire | 0.09 | [-5.02, 4.84] |
|  | Natural disaster | 0.09 | [-8.26, 8.09] |
| Factor 3 | |  |  |
|  | Non-suicidal self-injury | 0.10 | [-5.03, 5.49] |
|  | Head injury | 0.14 | [-9.88, 11.05] |
|  | Knocked unconscious | -0.27 | [-30.97, 28.58] |
|  | Traumatic brain injury | -0.13 | [-5.91, 4.85] |
|  | **Operation** | **0.23** | **[-8.14, 9.47]** |
|  | Broken bones | 0.10 | [-16.20, 17.75] |
|  | Sprains | 0.03 | [-12.85, 14.24] |
|  | **Stitches** | **0.28** | **[-8.30, 9.96]** |
|  | **Other serious wounds** | **0.31** | **[-33.58, 36.87]** |
|  | Falls | 0.02 | [-17.39, 19.47] |
|  | Burns | 0.16 | [-64.10, 69.04] |
|  | Bruises | -0.12 | [-20.26, 22.40] |
|  | **Broken teeth** | **0.22** | **[-13.39, 15.78]** |
|  | **Animal bites** | **0.78** | **[-26.78, 31.65]** |
|  | Threatened with death | 0.08 | [-9.57, 9.58] |
|  | Witnessed violence | -0.25 | [-8.71, 7.41] |
|  | Experienced violence | -0.85 | [-48.62, 42.40] |
|  | Exposure to violence | 0.07 | [-16.55, 15.75] |
|  | Sexual assault | -0.14 | [-31.67, 28.94] |
|  | Car accident | 0.05 | [-7.09, 7.50] |
|  | Other significant accident | 0.15 | [-9.40, 10.27] |
|  | Fire | 0.11 | [-7.82, 7.21] |
|  | Natural disaster | 0.01 | [-31.85, 29.78] |
| Factor 4 | |  |  |
|  | Non-suicidal self-injury | -0.03 | [-11.20, 12.36] |
|  | Head injury | 0.17 | [-39.42, 43.92] |
|  | Knocked unconscious | 0.01 | [-14.04, 15.91] |
|  | Traumatic brain injury | 0.05 | [-31.39, 34.58] |
|  | Operation | -0.02 | [-12.04, 13.30] |
|  | **Broken bones** | **0.22** | **[-27.16, 30.14]** |
|  | **Sprains** | **0.35** | **[-22.64, 25.63]** |
|  |  | Factor Loading | 95% CI |
|  | Stitches | 0.07 | [-21.00, 23.60] |
|  | Other serious wounds | 0.14 | [-31.61, 35.28] |
|  | **Falls** | **0.69** | **[-37.85, 42.92]** |
|  | **Burns** | **0.29** | **[-36.48, 40.45]** |
|  | **Bruises** | **0.93** | **[-30.47, 34.80]** |
|  | Broken teeth | 0.03 | [-11.24, 12.22] |
|  | Animal bites | 0.00 | [-21.69, 24.24] |
|  | Threatened with death | 0.11 | [-21.78, 24.15] |
|  | Witnessed violence | 0.21 | [-12.24, 14.00] |
|  | Experienced violence | 0.25 | [-10.81, 10.66] |
|  | Exposure to violence | 0.05 | [-7.54, 8.58] |
|  | Sexual assault | -0.09 | [-8.04, 8.70] |
|  | Car accident | 0.06 | [-20.93, 23.24] |
|  | Other significant accident | 0.11 | [-31.56, 35.34] |
|  | Fire | -0.22 | [-9.26, 10.21] |
|  | Natural disaster | -0.19 | [-6.39, 6.03] |

*Note.* Indicators that were carried forward for confirmatory factor analysis are shown in bold font. *Abbreviations.* CI = confidence interval.

Table S10. Confirmatory factor analysis of painful and provocative events in the first split-half sample.

|  |  | Factor Loading | 95% CI |
| --- | --- | --- | --- |
| Factor 1 | |  |  |
|  | *Non-suicidal self-injury* | *0.20* | *[0.12, 0.28]* |
|  | Threatened with death | 0.75 | [0.64, 0.86] |
|  | Witnessed violence | 0.54 | [0.48, 0.61] |
|  | Experienced violence | 0.84 | [0.74, 0.93] |
|  | Exposure to violence | 0.67 | [0.57, 0.77] |
|  | Sexual assault | 0.61 | [0.50, 0.72] |
|  | Car accident | 0.50 | [0.40, 0.60] |
|  | Other significant accident | 0.65 | [0.56, 0.73] |
|  | Fire | 0.49 | [0.36, 0.62] |
|  | Natural disaster | 0.44 | [0.31, 0.56] |
| Factor 2 | |  |  |
|  | Head injury | 0.80 | [0.75, 0.84] |
|  | Knocked unconscious | 0.97 | [0.92, 1.02] |
|  | Traumatic brain injury | 0.89 | [0.85, 0.93] |
| Factor 3 | |  |  |
|  | *Operation* | *0.14* | *[0.08, 0.19]* |
|  | Stitches | 0.32 | [0.27, 0.37] |
|  | Other serious wounds | 0.39 | [0.32, 0.46] |
|  | *Broken teeth* | *0.15* | *[0.07, 0.22]* |
|  | *Animal bites* | *0.28* | *[0.19, 0.37]* |
| Factor 4 | |  |  |
|  | Broken bones | 0.39 | [0.34, 0.44] |
|  | Sprains | 0.44 | [0.38, 0.49] |
|  | Falls | 0.83 | [0.78, 0.88] |
|  | *Burns* | *0.27* | *[0.18, 0.35]* |
|  | Bruises | 0.59 | [0.54, 0.64] |

*Note.* Indicators with factor loadings less than 0.30 are shown in italics. *Abbreviations.* CI = confidence interval.

Table S11. Correlations between the primary study variables in European ancestry individuals.

|  | **1** | **2** | **3** | **4** | **5** | **6** | **7** | **8** | **9** | **10** | **11** | **12** | **13** | **14** | **15** | **16** | **17** | **18** | **19** | **20** |
| --- | --- | --- | --- | --- | --- | --- | --- | --- | --- | --- | --- | --- | --- | --- | --- | --- | --- | --- | --- | --- |
| 1. PGS | 1 |  |  |  |  |  |  |  |  |  |  |  |  |  |  |  |  |  |  |  |
| 2. FHD | .07 | 1 |  |  |  |  |  |  |  |  |  |  |  |  |  |  |  |  |  |  |
| 3. Low conscientiousness | .02 | .05 | 1 |  |  |  |  |  |  |  |  |  |  |  |  |  |  |  |  |  |
| 4. Urgency | .04 | .06 | .28 | 1 |  |  |  |  |  |  |  |  |  |  |  |  |  |  |  |  |
| 5. Delay discounting | -.01 | .01 | .02 | .05 | 1 |  |  |  |  |  |  |  |  |  |  |  |  |  |  |  |
| 6. SSRT | -.01 | -.01 | -.05 | -.04 | .03 | 1 |  |  |  |  |  |  |  |  |  |  |  |  |  |  |
| 7. Sensation-seeking | .00 | .00 | .02 | .18 | -.01 | -.01 | 1 |  |  |  |  |  |  |  |  |  |  |  |  |  |
| 8. Traumatic events | .06 | .15 | .01 | .04 | .03 | .00 | -.01 | 1 |  |  |  |  |  |  |  |  |  |  |  |  |
| 9. Head injuries | .00 | .02 | .01 | .02 | .01 | .01 | .06 | .05 | 1 |  |  |  |  |  |  |  |  |  |  |  |
| 10. Serious wounds | .01 | .01 | .03 | .01 | .04 | -.02 | .03 | .14 | .09 | 1 |  |  |  |  |  |  |  |  |  |  |
| 11. Minor injuries | .02 | .04 | .02 | .02 | .02 | -.01 | .03 | .16 | .15 | .14 | 1 |  |  |  |  |  |  |  |  |  |
| 12. NSSI | .07 | .08 | .20 | .25 | .04 | -.01 | .01 | .05 | .01 | .04 | .00 | 1 |  |  |  |  |  |  |  |  |
| 13. Operations | .03 | .03 | .01 | .03 | .00 | -.02 | .00 | .05 | .02 | .07 | .08 | .08 | 1 |  |  |  |  |  |  |  |
| 14. SA | .14 | .19 | .13 | .23 | .03 | -.06 | .01 | .12 | -.03 | -.09 | .05 | .53 | .09 | 1 |  |  |  |  |  |  |
| 15. Sex | .03 | -.01 | -.14 | -.11 | -.11 | .02 | -.15 | .00 | -.03 | -.16 | .01 | -.12 | -.15 | .06 | 1 |  |  |  |  |  |
| 16. Age | .00 | .00 | -.03 | -.05 | -.01 | .12 | .04 | .00 | .03 | .03 | .06 | -.02 | .01 | .05 | -.04 | 1 |  |  |  |  |
| 17. Parental education | -.16 | -.17 | -.05 | -.13 | -.03 | .07 | .06 | -.15 | -.02 | -.05 | -.07 | -.10 | -.05 | -.13 | .01 | -.01 | 1 |  |  |  |
| 18. Income | -.08 | -.18 | -.09 | -.09 | .00 | .06 | .04 | -.18 | .00 | -.02 | -.02 | -.10 | .00 | -.15 | -.02 | .05 | .64 | 1 |  |  |
| 19. Financial difficulties | .04 | .17 | .05 | .07 | .01 | -.07 | -.01 | .17 | .03 | .05 | .07 | .13 | .04 | .11 | -.01 | -.03 | -.28 | -.40 | 1 |  |
| 20. Depressive symptoms | .06 | .06 | .06 | .13 | .02 | .00 | .02 | .03 | .03 | .01 | .03 | .15 | .03 | .13 | -.01 | .02 | -.07 | -.09 | .04 | 1 |

*Note.* Polyserial correlations were computed between continuous and ordinal variables, and polychoric correlations were computed between ordinal variables. The remaining correlation coefficients are Pearson product-moment correlations. *Abbreviations.* PGS = polygenic scores; FHD = family history scores; SSRT = stop-signal reaction time; NSSI = non-suicidal self-injury; SA = suicide attempt.

Table S12. Correlations between the primary study variables in African ancestry individuals.

|  | **1** | **2** | **3** | **4** | **5** | **6** | **7** | **8** | **9** | **10** | **11** | **12** | **13** | **14** | **15** | **16** | **17** | **18** | **19** | **20** |
| --- | --- | --- | --- | --- | --- | --- | --- | --- | --- | --- | --- | --- | --- | --- | --- | --- | --- | --- | --- | --- |
| 1. PGS | 1 |  |  |  |  |  |  |  |  |  |  |  |  |  |  |  |  |  |  |  |
| 2. FHD | -.06 | 1 |  |  |  |  |  |  |  |  |  |  |  |  |  |  |  |  |  |  |
| 3. Low conscientiousness | -.04 | .04 | 1 |  |  |  |  |  |  |  |  |  |  |  |  |  |  |  |  |  |
| 4. Urgency | -.02 | .00 | .17 | 1 |  |  |  |  |  |  |  |  |  |  |  |  |  |  |  |  |
| 5. Delay discounting | .00 | .01 | .04 | .02 | 1 |  |  |  |  |  |  |  |  |  |  |  |  |  |  |  |
| 6. SSRT | .01 | .02 | .00 | -.10 | .07 | 1 |  |  |  |  |  |  |  |  |  |  |  |  |  |  |
| 7. Sensation-seeking | -.01 | .01 | -.10 | .24 | .03 | -.05 | 1 |  |  |  |  |  |  |  |  |  |  |  |  |  |
| 8. Traumatic events | -.07 | .16 | .04 | .06 | -.01 | .01 | .08 | 1 |  |  |  |  |  |  |  |  |  |  |  |  |
| 9. Head injuries | -.04 | .12 | .01 | .02 | -.01 | .00 | -.01 | .11 | 1 |  |  |  |  |  |  |  |  |  |  |  |
| 10. Serious wounds | -.03 | .02 | .04 | -.02 | -.05 | .00 | .05 | .17 | .07 | 1 |  |  |  |  |  |  |  |  |  |  |
| 11. Minor injuries | -.02 | .03 | -.02 | -.03 | -.01 | .00 | .04 | .12 | .17 | .21 | 1 |  |  |  |  |  |  |  |  |  |
| 12. NSSI | -.10 | .08 | .15 | .24 | -.08 | -.06 | .02 | .14 | .00 | .08 | .02 | 1 |  |  |  |  |  |  |  |  |
| 13. Operations | -.04 | .09 | -.01 | -.03 | .00 | .02 | .01 | .11 | .08 | .17 | .14 | .16 | 1 |  |  |  |  |  |  |  |
| 14. SA | .06 | .09 | .11 | .11 | -.19 | -.12 | -.07 | .13 | -.04 | .12 | -.16 | .38 | .18 | 1 |  |  |  |  |  |  |
| 15. Sex | .01 | -.07 | -.13 | -.13 | -.06 | -.04 | -.16 | -.02 | -.13 | -.06 | -.01 | -.08 | -.05 | .20 | 1 |  |  |  |  |  |
| 16. Age | .04 | .03 | .01 | .01 | .07 | .15 | .01 | -.01 | .06 | -.01 | .02 | -.08 | -.02 | -.04 | .00 | 1 |  |  |  |  |
| 17. Parental education | -.10 | .00 | .03 | -.11 | -.04 | .07 | .01 | .03 | .14 | -.01 | .04 | .08 | .22 | -.04 | .01 | .06 | 1 |  |  |  |
| 18. Income | -.14 | -.08 | .02 | -.13 | .00 | .02 | -.01 | -.06 | .02 | -.02 | -.02 | .02 | .19 | -.07 | .06 | .00 | .74 | 1 |  |  |
| 19. Financial difficulties | .00 | .13 | .04 | .06 | .00 | .00 | -.02 | .14 | .06 | .04 | .07 | .08 | .01 | .03 | -.02 | .01 | -.15 | -.41 | 1 |  |
| 20. Depressive symptoms | .00 | .02 | .03 | .13 | -.01 | .02 | .06 | .06 | -.01 | .01 | .03 | .20 | .01 | .08 | -.04 | .03 | -.03 | -.03 | .00 |  |

*Note.* Polyserial correlations were computed between continuous and ordinal variables, and polychoric correlations were computed between ordinal variables. The remaining correlation coefficients are Pearson product-moment correlations. *Abbreviations.* PGS = polygenic scores; FHD = family history scores; SSRT = stop-signal reaction time; NSSI = non-suicidal self-injury; SA = suicide attempt.

# Table S13. Mediation model parameter estimates in European ancestry individuals with suicidal ideation.

|  | **Traumatic Events**  $\beta$ [95% CI] | **Head Injuries**  $\beta$ [95% CI] | **Serious Wounds**  $\beta$ [95% CI] | **Minor Injuries**  $\beta$ [95% CI] | **NSSI**  $\beta$ [95% CI] | **Operations**  $\beta$ [95% CI] |
| --- | --- | --- | --- | --- | --- | --- |
| **Low Conscientiousness** |  |  |  |  |  |  |
| IMP ~ PGS | 0.03 [-0.39, 0.44] | 0.03 [-0.31, 0.36] | 0.03 [-0.32, 0.37] | 0.03 [-0.32, 0.37] | 0.03 [-0.31, 0.36] | 0.03 [-0.31, 0.37] |
| IMP ~ FHD | 0.19 [-0.24, 0.63] | 0.19 [-0.16, 0.55] | 0.19 [-0.17, 0.55] | 0.19 [-0.17, 0.56] | 0.19 [-0.16, 0.55] | 0.19 [-0.16, 0.55] |
| PPE ~ PGS | 0.05 [-0.84, 0.94] | 0.09 [-0.30, 0.47] | 0.14 [-0.88, 1.17] | 0.52 [-0.83, 1.86] | 0.07 [-0.23, 0.38] | 0.05 [-0.30, 0.40] |
| PPE ~ FHD | 0.56 [-0.15, 1.27] | 0.06 [-0.29, 0.40] | 0.15 [-1.02, 1.32] | 0.02 [-1.03, 1.08] | -0.06 [-0.38, 0.26] | 0.05 [-0.30, 0.40] |
| PPE ~ IMP | -0.19 [-0.66, 0.29] | 0.00 [-0.18, 0.18] | 0.01 [-0.65, 0.68] | -0.09 [-0.77, 0.59] | 0.12 [-0.08, 0.32] | 0.02 [-0.20, 0.24] |
| SA ~ PGS | 0.08 [-0.57, 0.72] | 0.09 [-0.41, 0.59] | 0.12 [-0.42, 0.66] | -0.03 [-0.58, 0.52] | 0.06 [-0.42, 0.54] | 0.08 [-0.42, 0.58] |
| SA ~ FHD | 0.07 [-0.59, 0.73] | 0.17 [-0.34, 0.69] | 0.21 [-0.38, 0.80] | 0.17 [-0.40, 0.74] | 0.20 [-0.31, 0.71] | 0.17 [-0.35, 0.68] |
| SA ~ IMP | -0.20 [-0.48, 0.07] | -0.24 [-0.43, -0.04] | -0.23 [-0.49, 0.02] | -0.21 [-0.46, 0.04] | -0.29 [-0.51, -0.06] | -0.24 [-0.43, -0.04] |
| SA ~ PPE | **0.19 [0.06, 0.31]** | -0.03 [-0.37, 0.30] | -0.23 [-0.39, -0.07] | 0.23 [0.05, 0.41] | 0.42 [-0.06, 0.91] | 0.10 [-0.41, 0.62] |
| **Urgency** |  |  |  |  |  |  |
| IMP ~ PGS | 0.03 [-0.32, 0.39] | 0.03 [-0.16, 0.23] | 0.03 [-0.18, 0.25] | 0.03 [-0.18, 0.25] | 0.03 [-0.15, 0.22] | 0.03 [-0.15, 0.22] |
| IMP ~ FHD | 0.08 [-0.30, 0.45] | 0.08 [-0.13, 0.28] | 0.08 [-0.14, 0.30] | 0.08 [-0.15, 0.30] | 0.08 [-0.12, 0.27] | 0.08 [-0.12, 0.27] |
| PPE ~ PGS | 0.05 [-0.75, 0.85] | 0.09 [-0.14, 0.32] | 0.14 [-0.51, 0.79] | 0.51 [-0.33, 1.35] | 0.07 [-0.11, 0.25] | 0.05 [-0.15, 0.25] |
| PPE ~ FHD | 0.53 [-0.10, 1.16] | 0.06 [-0.14, 0.26] | 0.14 [-0.59, 0.87] | 0.00 [-0.66, 0.66] | -0.04 [-0.22, 0.14] | 0.05 [-0.15, 0.25] |
| PPE ~ IMP | -0.13 [-0.60, 0.35] | -0.10 [-0.23, 0.03] | 0.19 [-0.31, 0.70] | 0.09 [-0.38, 0.57] | 0.14 [0.02, 0.26] | 0.06 [-0.07, 0.19] |
| SA ~ PGS | 0.07 [-0.48, 0.62] | 0.08 [-0.21, 0.36] | 0.11 [-0.21, 0.44] | -0.04 [-0.37, 0.29] | 0.06 [-0.20, 0.32] | 0.08 [-0.19, 0.34] |
| SA ~ FHD | 0.00 [-0.57, 0.57] | 0.11 [-0.19, 0.41] | 0.14 [-0.22, 0.50] | 0.11 [-0.24, 0.45] | 0.12 [-0.16, 0.40] | 0.10 [-0.17, 0.38] |
| SA ~ IMP | 0.27 [-0.10, 0.63] | 0.24 [0.06, 0.42] | 0.28 [0.06, 0.51] | 0.22 [0.00, 0.44] | 0.20 [0.03, 0.37] | 0.24 [0.06, 0.41] |
| SA ~ PPE | **0.21 [0.11, 0.31]** | -0.01 [-0.20, 0.19] | **-0.24 [-0.34, -0.14]** | **0.23 [0.12, 0.34]** | 0.31 [0.05, 0.56] | 0.07 [-0.21, 0.34] |
| **Delay Discounting** |  |  |  |  |  |  |
| IMP ~ PGS | -0.08 [-0.82, 0.65] | -0.08 [-0.65, 0.49] | -0.08 [-0.66, 0.50] | -0.08 [-0.66, 0.49] | -0.08 [-0.66, 0.49] | -0.08 [-0.65, 0.49] |
| IMP ~ FHD | 0.17 [-0.61, 0.96] | 0.17 [-0.43, 0.78] | 0.17 [-0.44, 0.79] | 0.17 [-0.44, 0.79] | 0.17 [-0.44, 0.79] | 0.17 [-0.43, 0.78] |
| PPE ~ PGS | 0.05 [-0.92, 1.01] | 0.08 [-0.31, 0.48] | 0.15 [-0.90, 1.20] | 0.52 [-0.84, 1.89] | 0.08 [-0.25, 0.40] | 0.05 [-0.31, 0.41] |
| PPE ~ FHD | 0.52 [-0.24, 1.28] | 0.06 [-0.29, 0.41] | 0.15 [-1.04, 1.34] | -0.02 [-1.07, 1.03] | -0.03 [-0.36, 0.30] | 0.05 [-0.31, 0.41] |
| PPE ~ IMP | 0.01 [-0.23, 0.24] | -0.02 [-0.13, 0.09] | 0.01 [-0.39, 0.42] | 0.14 [-0.31, 0.60] | -0.02 [-0.15, 0.11] | 0.04 [-0.10, 0.17] |
| SA ~ PGS | 0.09 [-0.60, 0.78] | 0.10 [-0.41, 0.61] | 0.13 [-0.41, 0.68] | -0.02 [-0.57, 0.53] | 0.07 [-0.42, 0.56] | 0.10 [-0.41, 0.60] |
| SA ~ FHD | 0.00 [-0.73, 0.72] | 0.10 [-0.44, 0.64] | 0.13 [-0.47, 0.73] | 0.10 [-0.48, 0.68] | 0.11 [-0.42, 0.64] | 0.10 [-0.44, 0.63] |
| SA ~ IMP | 0.15 [-0.02, 0.33] | 0.16 [0.03, 0.28] | 0.16 [0.00, 0.32] | 0.12 [-0.04, 0.29] | 0.16 [0.03, 0.30] | 0.15 [0.03, 0.28] |
|  | **Traumatic Events**  $\beta$ [95% CI] | **Head Injuries**  $\beta$ [95% CI] | **Serious Wounds**  $\beta$ [95% CI] | **Minor Injuries**  $\beta$ [95% CI] | **NSSI**  $\beta$ [95% CI] | **Operations**  $\beta$ [95% CI] |
| SA ~ PPE | **0.20 [0.08, 0.32]** | -0.02 [-0.38, 0.33] | -0.23 [-0.40, -0.07] | 0.22 [0.04, 0.41] | 0.37 [-0.13, 0.88] | 0.05 [-0.49, 0.60] |
| **Low Response Inhibition** | |  |  |  |  |  |
| IMP ~ PGS | -0.03 [-0.32, 0.27] | -0.03 [-0.23, 0.18] | -0.03 [-0.24, 0.19] | -0.03 [-0.24, 0.19] | -0.03 [-0.23, 0.18] | -0.03 [-0.23, 0.18] |
| IMP ~ FHD | 0.01 [-0.36, 0.38] | 0.01 [-0.25, 0.26] | 0.01 [-0.26, 0.28] | 0.01 [-0.26, 0.27] | 0.01 [-0.25, 0.26] | 0.01 [-0.25, 0.26] |
| PPE ~ PGS | 0.04 [-0.83, 0.92] | 0.09 [-0.23, 0.41] | 0.14 [-0.74, 1.02] | 0.52 [-0.62, 1.66] | 0.08 [-0.18, 0.34] | 0.05 [-0.24, 0.35] |
| PPE ~ FHD | 0.52 [-0.17, 1.21] | 0.06 [-0.23, 0.34] | 0.15 [-0.85, 1.15] | 0.00 [-0.88, 0.89] | -0.03 [-0.30, 0.23] | 0.05 [-0.24, 0.34] |
| PPE ~ IMP | -0.11 [-0.80, 0.59] | 0.04 [-0.17, 0.26] | -0.11 [-0.98, 0.76] | 0.10 [-0.68, 0.89] | -0.01 [-0.25, 0.22] | 0.07 [-0.17, 0.32] |
| SA ~ PGS | 0.10 [-0.55, 0.74] | 0.11 [-0.31, 0.53] | 0.14 [-0.34, 0.61] | -0.01 [-0.48, 0.46] | 0.08 [-0.33, 0.48] | 0.10 [-0.32, 0.53] |
| SA ~ FHD | 0.02 [-0.67, 0.70] | 0.13 [-0.32, 0.57] | 0.16 [-0.35, 0.66] | 0.12 [-0.38, 0.63] | 0.14 [-0.29, 0.57] | 0.12 [-0.32, 0.57] |
| SA ~ IMP | **0.88 [0.53, 1.22]** | **0.85 [0.67, 1.04]** | **0.83 [0.55, 1.10]** | **0.83 [0.57, 1.09]** | **0.86 [0.66, 1.05]** | **0.85 [0.67, 1.03]** |
| SA ~ PPE | **0.21 [0.07, 0.35]** | -0.06 [-0.35, 0.23] | **-0.23 [-0.37, -0.09]** | 0.22 [0.07, 0.38] | 0.36 [-0.04, 0.76] | 0.03 [-0.41, 0.47] |
| **Sensation-Seeking** |  |  |  |  |  |  |
| IMP ~ PGS | 0.00 [-0.28, 0.28] | 0.00 [-0.18, 0.19] | 0.00 [-0.19, 0.20] | 0.00 [-0.20, 0.20] | 0.00 [-0.18, 0.19] | 0.00 [-0.18, 0.18] |
| IMP ~ FHD | -0.02 [-0.33, 0.28] | -0.02 [-0.23, 0.18] | -0.02 [-0.24, 0.19] | -0.02 [-0.24, 0.20] | -0.02 [-0.23, 0.18] | -0.02 [-0.22, 0.18] |
| PPE ~ PGS | 0.05 [-0.78, 0.87] | 0.09 [-0.21, 0.38] | 0.15 [-0.65, 0.95] | 0.51 [-0.54, 1.56] | 0.08 [-0.16, 0.32] | 0.05 [-0.21, 0.32] |
| PPE ~ FHD | 0.52 [-0.13, 1.16] | 0.06 [-0.20, 0.31] | 0.16 [-0.75, 1.07] | 0.00 [-0.81, 0.82] | -0.03 [-0.28, 0.21] | 0.06 [-0.21, 0.32] |
| PPE ~ IMP | -0.11 [-0.79, 0.58] | 0.00 [-0.20, 0.21] | 0.14 [-0.65, 0.94] | 0.08 [-0.67, 0.84] | -0.06 [-0.29, 0.16] | 0.08 [-0.16, 0.32] |
| SA ~ PGS | 0.07 [-0.52, 0.66] | 0.08 [-0.29, 0.46] | 0.12 [-0.30, 0.53] | -0.03 [-0.46, 0.39] | 0.05 [-0.30, 0.41] | 0.08 [-0.29, 0.45] |
| SA ~ FHD | 0.04 [-0.58, 0.65] | 0.14 [-0.26, 0.54] | 0.18 [-0.29, 0.64] | 0.14 [-0.31, 0.59] | 0.15 [-0.24, 0.54] | 0.14 [-0.26, 0.53] |
| SA ~ IMP | 0.57 [0.18, 0.96] | **0.55 [0.32, 0.77]** | **0.58 [0.28, 0.88]** | **0.53 [0.23, 0.82]** | **0.57 [0.33, 0.81]** | **0.54 [0.32, 0.77]** |
| SA ~ PPE | **0.21 [0.09, 0.33]** | -0.04 [-0.29, 0.22] | **-0.24 [-0.36, -0.11]** | **0.23 [0.09, 0.37]** | 0.39 [0.04, 0.74] | 0.05 [-0.34, 0.44] |

*Note.* Results are presented as beta values with 95% confidence intervals. Statistically significant parameter estimates (FDR-corrected *p* < .05) are shown in bold font. Due to space limitations, only the path coefficients of central interest are shown. CI = confidence interval; IMP = impulsivity; PGS = polygenic scores; FHD = family history density scores; PPE = painful and provocative event; SA = suicide attempt.

# Table S14. Mediation model parameter estimates in African ancestry individuals with suicidal ideation.

|  | **Traumatic Events**  $\beta$ [95% CI] | **Head Injuries**  $\beta$ [95% CI] | **Serious Wounds**  $\beta$ [95% CI] | **Minor Injuries**  $\beta$ [95% CI] | **NSSI**  $\beta$ [95% CI] | **Operations**  $\beta$ [95% CI] |
| --- | --- | --- | --- | --- | --- | --- |
| **Low Conscientiousness** |  |  |  |  |  |  |
| IMP ~ PGS | -0.13 [-0.36, 0.11] | -0.13 [-0.36, 0.10] | -0.13 [-0.36, 0.11] | -0.13 [-0.36, 0.11] | -0.13 [-0.36, 0.10] | -0.13 [-0.38, 0.11] |
| IMP ~ FHD | 0.08 [-0.15, 0.32] | 0.08 [-0.15, 0.32] | 0.08 [-0.15, 0.32] | 0.08 [-0.15, 0.32] | 0.08 [-0.15, 0.32] | 0.07 [-0.17, 0.31] |
| PPE ~ PGS | -0.21 [-0.65, 0.23] | 0.16 [-0.41, 0.74] | -0.23 [-0.84, 0.39] | 0.27 [-0.37, 0.91] | -0.24 [-0.46, -0.02] | 0.10 [-0.13, 0.33] |
| PPE ~ FHD | 0.19 [-0.31, 0.69] | **0.75 [0.38, 1.12]** | -0.08 [-0.86, 0.70] | 0.27 [-0.41, 0.96] | -0.03 [-0.23, 0.16] | 0.11 [-0.13, 0.35] |
| PPE ~ IMP | -0.08 [-0.33, 0.18] | -0.07 [-0.30, 0.17] | 0.00 [-0.36, 0.36] | 0.15 [-0.27, 0.57] | 0.16 [0.04, 0.28] | 0.06 [-0.05, 0.16] |
| SA ~ PGS | 0.13 [-0.19, 0.45] | 0.14 [-0.19, 0.47] | 0.13 [-0.19, 0.45] | 0.15 [-0.17, 0.47] | 0.18 [-0.13, 0.49] | 0.11 [-0.21, 0.44] |
| SA ~ FHD | 0.00 [-0.30, 0.31] | 0.09 [-0.23, 0.41] | 0.01 [-0.29, 0.32] | 0.04 [-0.27, 0.34] | 0.02 [-0.27, 0.31] | -0.01 [-0.31, 0.29] |
| SA ~ IMP | 0.09 [-0.05, 0.23] | 0.08 [-0.07, 0.22] | 0.08 [-0.06, 0.22] | 0.10 [-0.04, 0.24] | 0.04 [-0.10, 0.19] | 0.07 [-0.07, 0.21] |
| SA ~ PPE | 0.04 [-0.03, 0.12] | -0.10 [-0.25, 0.04] | 0.04 [0.00, 0.08] | **-0.10 [-0.15, -0.05]** | 0.24 [0.00, 0.47] | 0.24 [-0.01, 0.49] |
| **Urgency** |  |  |  |  |  |  |
| IMP ~ PGS | -0.08 [-0.27, 0.10] | -0.08 [-0.27, 0.10] | -0.08 [-0.27, 0.11] | -0.08 [-0.27, 0.10] | -0.08 [-0.27, 0.10] | -0.10 [-0.29, 0.09] |
| IMP ~ FHD | -0.03 [-0.23, 0.17] | -0.03 [-0.23, 0.17] | -0.03 [-0.23, 0.17] | -0.03 [-0.23, 0.17] | -0.03 [-0.22, 0.17] | -0.05 [-0.24, 0.15] |
| PPE ~ PGS | -0.20 [-0.64, 0.23] | 0.17 [-0.40, 0.74] | -0.27 [-0.88, 0.34] | 0.24 [-0.39, 0.87] | -0.24 [-0.46, -0.02] | 0.09 [-0.14, 0.32] |
| PPE ~ FHD | 0.18 [-0.32, 0.68] | **0.74 [0.37, 1.11]** | -0.09 [-0.84, 0.66] | 0.28 [-0.40, 0.97] | -0.02 [-0.21, 0.18] | 0.11 [-0.12, 0.34] |
| PPE ~ IMP | -0.08 [-0.35, 0.19] | -0.01 [-0.31, 0.28] | -0.48 [-0.99, 0.04] | -0.11 [-0.62, 0.39] | 0.16 [0.02, 0.31] | -0.02 [-0.19, 0.15] |
| SA ~ PGS | 0.12 [-0.21, 0.44] | 0.13 [-0.20, 0.45] | 0.12 [-0.21, 0.44] | 0.13 [-0.19, 0.45] | 0.18 [-0.14, 0.49] | 0.09 [-0.22, 0.40] |
| SA ~ FHD | 0.01 [-0.29, 0.31] | 0.09 [-0.22, 0.41] | 0.02 [-0.28, 0.32] | 0.04 [-0.26, 0.34] | 0.02 [-0.27, 0.31] | -0.03 [-0.32, 0.27] |
| SA ~ IMP | -0.06 [-0.26, 0.14] | -0.06 [-0.27, 0.14] | -0.04 [-0.25, 0.16] | -0.07 [-0.28, 0.13] | -0.11 [-0.31, 0.09] | -0.07 [-0.27, 0.12] |
| SA ~ PPE | 0.04 [-0.04, 0.11] | -0.10 [-0.25, 0.04] | 0.04 [-0.01, 0.08] | **-0.10 [-0.14, -0.05]** | 0.28 [0.05, 0.51] | 0.25 [0.01, 0.49] |
| **Delay Discounting** |  |  |  |  |  |  |
| IMP ~ PGS | -0.04 [-0.44, 0.36] | -0.04 [-0.43, 0.36] | -0.04 [-0.44, 0.36] | -0.04 [-0.44, 0.36] | -0.04 [-0.43, 0.36] | -0.02 [-0.48, 0.44] |
| IMP ~ FHD | -0.25 [-0.59, 0.09] | -0.25 [-0.59, 0.09] | -0.25 [-0.59, 0.09] | -0.25 [-0.59, 0.09] | -0.25 [-0.58, 0.08] | -0.24 [-0.63, 0.14] |
| PPE ~ PGS | -0.20 [-0.64, 0.25] | 0.17 [-0.41, 0.75] | -0.23 [-0.85, 0.39] | 0.25 [-0.40, 0.90] | -0.26 [-0.48, -0.03] | 0.09 [-0.19, 0.37] |
| PPE ~ FHD | 0.18 [-0.33, 0.69] | **0.75 [0.36, 1.13]** | -0.09 [-0.89, 0.70] | 0.29 [-0.42, 0.99] | -0.01 [-0.22, 0.20] | 0.09 [-0.20, 0.37] |
| PPE ~ IMP | -0.03 [-0.16, 0.11] | 0.01 [-0.12, 0.13] | -0.07 [-0.33, 0.19] | 0.00 [-0.25, 0.26] | 0.03 [-0.05, 0.11] | -0.04 [-0.16, 0.09] |
| SA ~ PGS | 0.12 [-0.21, 0.44] | 0.13 [-0.20, 0.46] | 0.12 [-0.20, 0.44] | 0.13 [-0.19, 0.46] | 0.18 [-0.14, 0.49] | 0.09 [-0.95, 1.12] |
| SA ~ FHD | -0.02 [-0.33, 0.29] | 0.07 [-0.26, 0.39] | -0.01 [-0.32, 0.31] | 0.02 [-0.29, 0.33] | -0.01 [-0.31, 0.29] | -0.04 [-0.41, 0.33] |
| SA ~ IMP | -0.12 [-0.22, -0.03] | -0.12 [-0.22, -0.03] | -0.12 [-0.22, -0.03] | -0.12 [-0.22, -0.03] | -0.13 [-0.23, -0.03] | -0.09 [-5.06, 4.87] |
|  | **Traumatic Events**  $\beta$ [95% CI] | **Head Injuries**  $\beta$ [95% CI] | **Serious Wounds**  $\beta$ [95% CI] | **Minor Injuries**  $\beta$ [95% CI] | **NSSI**  $\beta$ [95% CI] | **Operations**  $\beta$ [95% CI] |
| SA ~ PPE | 0.04 [-0.04, 0.11] | -0.10 [-0.25, 0.04] | 0.04 [-0.01, 0.08] | **-0.09 [-0.14, -0.05]** | 0.27 [0.04, 0.50] | 0.37 [-11.0, 11.7] |
| **Low Response Inhibition** | |  |  |  |  |  |
| IMP ~ PGS | 0.13 [-0.03, 0.29] | 0.13 [-0.03, 0.29] | 0.13 [-0.03, 0.29] | 0.13 [-0.03, 0.29] | 0.13 [-0.03, 0.29] | 0.13 [-0.03, 0.29] |
| IMP ~ FHD | **0.19 [0.06, 0.33]** | 0.19 [0.06, 0.33] | 0.19 [0.06, 0.33] | 0.19 [0.06, 0.33] | **0.19 [0.06, 0.33]** | **0.20 [0.06, 0.33]** |
| PPE ~ PGS | -0.24 [-0.66, 0.18] | 0.10 [-0.46, 0.65] | -0.25 [-0.85, 0.35] | 0.22 [-0.40, 0.83] | -0.26 [-0.48, -0.04] | 0.12 [-0.11, 0.34] |
| PPE ~ FHD | 0.12 [-0.38, 0.62] | **0.63 [0.27, 0.99]** | -0.12 [-0.89, 0.65] | 0.24 [-0.43, 0.91] | -0.03 [-0.23, 0.18] | 0.14 [-0.09, 0.36] |
| PPE ~ IMP | 0.35 [-0.06, 0.76] | **0.58 [0.28, 0.88]** | 0.19 [-0.38, 0.77] | 0.25 [-0.34, 0.83] | 0.03 [-0.16, 0.21] | -0.18 [-0.41, 0.04] |
| SA ~ PGS | 0.11 [-0.21, 0.42] | 0.11 [-0.22, 0.44] | 0.11 [-0.21, 0.43] | 0.12 [-0.20, 0.44] | 0.17 [-0.14, 0.47] | 0.07 [-0.24, 0.38] |
| SA ~ FHD | -0.01 [-0.30, 0.29] | 0.08 [-0.24, 0.39] | 0.00 [-0.30, 0.30] | 0.02 [-0.27, 0.32] | 0.00 [-0.28, 0.29] | -0.04 [-0.33, 0.24] |
| SA ~ IMP | 0.09 [-0.14, 0.32] | 0.17 [-0.07, 0.42] | 0.09 [-0.13, 0.32] | 0.13 [-0.09, 0.34] | 0.10 [-0.14, 0.33] | 0.15 [-0.08, 0.39] |
| SA ~ PPE | 0.04 [-0.04, 0.11] | -0.12 [-0.27, 0.03] | 0.04 [0.00, 0.08] | **-0.10 [-0.14, -0.05]** | 0.25 [0.03, 0.47] | 0.28 [0.04, 0.51] |
| **Sensation-Seeking** |  |  |  |  |  |  |
| IMP ~ PGS | 0.04 [-0.11, 0.19] | 0.04 [-0.11, 0.19] | 0.04 [-0.12, 0.20] | 0.04 [-0.11, 0.19] | 0.04 [-0.11, 0.19] | 0.04 [-0.11, 0.19] |
| IMP ~ FHD | 0.00 [-0.16, 0.16] | 0.00 [-0.16, 0.16] | 0.00 [-0.16, 0.17] | 0.00 [-0.16, 0.16] | 0.00 [-0.16, 0.16] | 0.00 [-0.16, 0.16] |
| PPE ~ PGS | -0.20 [-0.62, 0.21] | 0.18 [-0.38, 0.73] | -0.22 [-0.82, 0.37] | 0.25 [-0.36, 0.87] | -0.25 [-0.47, -0.04] | 0.10 [-0.12, 0.32] |
| PPE ~ FHD | 0.18 [-0.29, 0.66] | **0.74 [0.39, 1.10]** | -0.08 [-0.83, 0.68] | 0.29 [-0.38, 0.95] | -0.02 [-0.21, 0.17] | 0.10 [-0.13, 0.34] |
| PPE ~ IMP | 0.21 [-0.21, 0.63] | -0.20 [-0.66, 0.27] | -0.07 [-0.58, 0.45] | -0.09 [-0.67, 0.48] | -0.11 [-0.30, 0.08] | -0.17 [-0.36, 0.01] |
| SA ~ PGS | 0.12 [-0.19, 0.43] | 0.13 [-0.18, 0.45] | 0.12 [-0.19, 0.44] | 0.14 [-0.17, 0.45] | 0.18 [-0.13, 0.48] | 0.09 [-0.21, 0.39] |
| SA ~ FHD | 0.01 [-0.28, 0.30] | 0.10 [-0.21, 0.40] | 0.02 [-0.28, 0.32] | 0.05 [-0.25, 0.34] | 0.02 [-0.26, 0.30] | -0.01 [-0.29, 0.27] |
| SA ~ IMP | -0.08 [-0.30, 0.14] | -0.09 [-0.30, 0.13] | -0.07 [-0.28, 0.15] | -0.08 [-0.28, 0.13] | -0.04 [-0.25, 0.17] | -0.02 [-0.24, 0.20] |
| SA ~ PPE | 0.04 [-0.03, 0.11] | -0.11 [-0.25, 0.03] | 0.04 [0.00, 0.08] | **-0.10 [-0.14, -0.05]** | 0.25 [0.03, 0.47] | 0.25 [0.02, 0.49] |

*Note.* Results are presented as beta values with 95% confidence intervals. Statistically significant parameter estimates (FDR-corrected *p* < .05) are shown in bold font. Due to space limitations, only the path coefficients of central interest are shown. CI = confidence interval; IMP = impulsivity; PGS = polygenic scores; FHD = family history density scores; PPE = painful and provocative event; SA = suicide attempt.

**
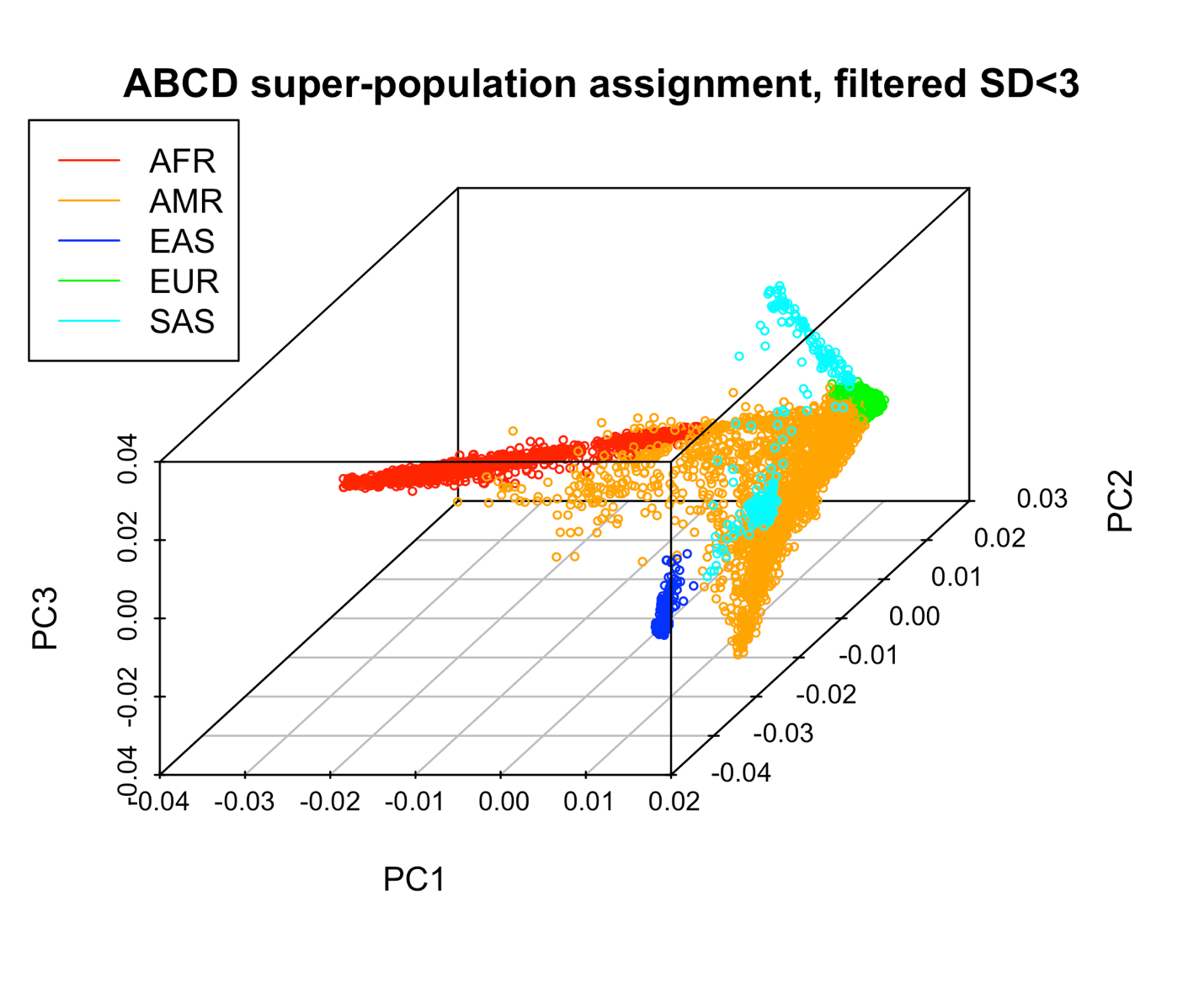
**

Figure S1. Assignment to super-populations in the Adolescent Brain Cognitive Development Study (ABCD). The first three principal components are plotted. Participants were assigned to one of five genetic-based super-populations based on the minimum Mahalanobis distance. Multivariate outliers (> 3 standard deviations from the super-population median) were removed. *Abbreviations.* AFR = African, AMR = admixed from the Americas, EAS = East Asian, EUR = European, SAS = South Asian, PC = principal component, SD = standard deviation.


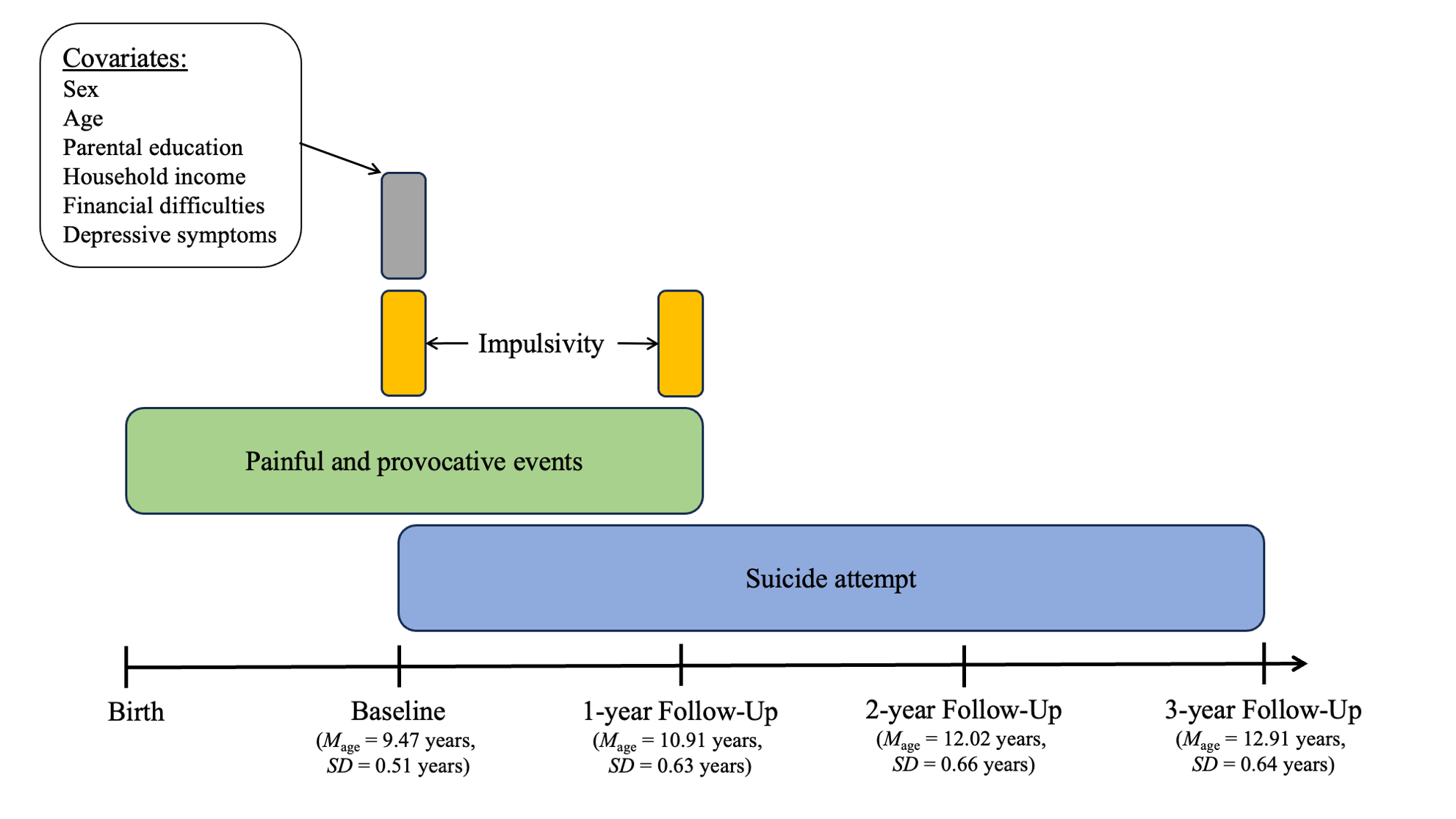


Figure S2. Diagrammatic representation of assessment timing. Covariates were measured during the baseline assessment. Measures of impulsivity and painful and provocative events (PPEs) were drawn from the baseline and 1-year follow-up assessments. Several PPE measures asked the adolescent and/or caregiver to report on the adolescent’s lifetime; as a result, these reports capture PPE exposure from birth through the 1-year follow-up assessment. Reports of SA were combined across follow-up assessments.
